# Systematic Review and Meta-Analysis of Endurance Exercise Training Protocols for Mice

**DOI:** 10.3389/fphys.2021.782695

**Published:** 2021-12-02

**Authors:** Michael P. Massett, Caitlyn Matejka, Hyoseon Kim

**Affiliations:** Department of Kinesiology and Sport Management, Texas Tech University, Lubbock, TX, United States

**Keywords:** inbred mice, treadmill running, sedentary, training responses, endurance exercise training

## Abstract

Inbred and genetically modified mice are frequently used to investigate the molecular mechanisms responsible for the beneficial adaptations to exercise training. However, published paradigms for exercise training in mice are variable, making comparisons across studies for training efficacy difficult. The purpose of this systematic review and meta-analysis was to characterize the diversity across published treadmill-based endurance exercise training protocols for mice and to identify training protocol parameters that moderate the adaptations to endurance exercise training in mice. Published studies were retrieved from PubMed and EMBASE and reviewed for the following inclusion criteria: inbred mice; inclusion of a sedentary group; and exercise training using a motorized treadmill. Fifty-eight articles met those inclusion criteria and also included a “classical” marker of training efficacy. Outcome measures included changes in exercise performance, $\dot{V}$O_2max_, skeletal muscle oxidative enzyme activity, blood lactate levels, or exercise-induced cardiac hypertrophy. The majority of studies were conducted using male mice. Approximately 48% of studies included all information regarding exercise training protocol parameters. Meta-analysis was performed using 105 distinct training groups (i.e., EX-SED pairs). Exercise training had a significant effect on training outcomes, but with high heterogeneity (Hedges’ *g*=1.70, 95% CI=1.47–1.94, Tau^2^=1.14, *I^2^*=80.4%, prediction interval=−0.43–3.84). Heterogeneity was partially explained by subgroup differences in treadmill incline, training duration, exercise performance test type, and outcome variable. Subsequent analyses were performed on subsets of studies based on training outcome, exercise performance, or biochemical markers. Exercise training significantly improved performance outcomes (Hedges’ *g*=1.85, 95% CI=1.55–2.15). Subgroup differences were observed for treadmill incline, training duration, and exercise performance test protocol on improvements in performance. Biochemical markers also changed significantly with training (Hedges’ *g*=1.62, 95% CI=1.14–2.11). Subgroup differences were observed for strain, sex, exercise session time, and training duration. These results demonstrate there is a high degree of heterogeneity across exercise training studies in mice. Training duration had the most significant impact on training outcome. However, the magnitude of the effect of exercise training varies based on the marker used to assess training efficacy.

## Introduction

Cardiovascular disease accounts for one in four deaths (~23%) in the United States (Murphy et al., 2021). Cancer, Alzheimer’s disease, diabetes, and hypertension also rank within the top 15 causes of death in the United States (Murphy et al., 2021). These chronic diseases have been linked to low levels of cardiorespiratory fitness (Defina et al., 2013; Zhang et al., 2014; Zaccardi et al., 2015; Sui et al., 2017; Robsahm et al., 2019; Lee, 2021). The Centers for Disease Control and Prevention and the American College of Sports Medicine currently recommend that individuals participate in moderate-intensity physical activity for 150 or more minutes per week for optimal health (Haskell et al., 2007; Garber et al., 2011). Improving cardiorespiratory fitness through increased physical activity can significantly reduce the risk of all-cause mortality (Blair et al., 1995; Brawner et al., 2017; Davidson et al., 2018). Although the majority of health benefits associated with high cardiorespiratory fitness are mediated by changes in traditional risk factors such as blood pressure, inflammatory markers, and blood lipids, roughly 40% of the beneficial effects of exercise cannot be explained by traditional risk factors (Mora et al., 2007; Joyner and Green, 2009). Furthermore, the cellular and molecular mechanisms underlying the salutary effects of exercise are not well understood. Therefore, inbred and genetically modified mice are frequently used to investigate the integrative physiological responses to exercise and the molecular mechanisms responsible for the beneficial adaptations to exercise training.

There are three commonly used paradigms for exercise training in rodents – swimming, voluntary wheel running, and “forced” wheel or treadmill running – and each has been used to study the molecular basis of responses to acute exercise and chronic exercise training. Treadmill running and wheel running induce adaptations in mice associated with endurance exercise training (Allen et al., 2001; Kemi et al., 2002; De Angelis et al., 2004; Waters et al., 2004; Massett and Berk, 2005; Chow et al., 2007). However, the two paradigms are inherently different (Poole et al., 2020) such that the correlation between treadmill running performance and voluntary wheel-running performance among mouse strains is nominal (Allen et al., 2001; Lightfoot et al., 2001, 2004). One advantage of treadmill running as an exercise paradigm is that the total amount of work performed among all mice can be established by the investigator through the selection of exercise testing and training parameters. Unlike for humans, there are no published well-accepted standards for exercise training paradigms or levels of activity required for optimal changes in exercise capacity or other training adaptations (Fuller and Thyfault, 2021). The published exercise testing and training paradigms are quite variable (Kemi et al., 2002; Billat et al., 2005; Hoydal et al., 2007; Marcaletti et al., 2011; Ayachi et al., 2016; Petrosino et al., 2016). Therefore, the purpose of this review was to characterize the variation in exercise training protocols in mice and determine key training parameters involved in adaptations to exercise training. This review focuses on treadmill running because the training parameters can be more easily quantified and any potential recommendations regarding these parameters could be incorporated into future research utilizing treadmill-based exercise training in mice.

## Materials and Methods

The protocol for systematic reviews of animal studies was used as a guide for this review and meta-analysis (de Vries et al., 2015).

The following terms were used to search PubMed and EMBASE databases: (((((((““Inbred Mouse Strains”” OR ““Inbred Strain of Mice”” OR ““Inbred Strain of Mouse”” OR ““Inbred Strains of Mice”” OR ““Mice, Inbred Strains””[MeSH Terms] OR ““Mice, Inbred Strains”” OR ““Mouse, Inbred Strain””) OR (““Mice””[MeSH Terms] OR ““Mice”” OR ““Mice, House”” OR ““Mice, Laboratory”” OR ““Mouse”” OR ““Mouse, House”” OR ““Mouse, Laboratory”” OR ““Mouse, Swiss”” OR ““Mus”” OR ““Mus domesticus”” OR ““Mus musculus”” OR ““Mus musculus domesticus”” OR ““Swiss Mice””))) NOT ((transgenic OR knockout OR db/db OR ob/ob OR mdx OR ApoE)))) AND (((((““Aerobic Exercise”” OR ““Exercise””[MeSH Terms] OR ““Exercise”” OR ““Exercise Training”” OR ““Exercise, Aerobic”” OR ““Exercise, Physical”” OR ““Physical Activity”” OR ““Running””[MeSH Terms] OR ““Running””))) OR (((““High-Intensity Intermittent Exercise”” OR ““High-Intensity Interval Training””[MeSH Terms] OR ““High-Intensity Interval Training”” OR ““Sprint Interval Training””)))))) AND treadmill) AND (sedentary OR control).” Additional abstracts were obtained from reference lists of potentially eligible articles. The search was competed in February 2020.

### Inclusion and Exclusion Criteria

Studies were included if they utilized inbred or wild-type mice of any strain divided into at least two groups: exercise training and sedentary control, the duration of the exercise training protocol was at least 1week, and the training was performed on a motor-driven treadmill. Studies also needed to include an outcome measure of training efficacy reported for both the exercise-trained and sedentary control groups. Acceptable outcome measures included assessment of exercise performance or oxygen consumption ($\dot{V}$O_2_), skeletal muscle oxidative enzymes (e.g., citrate synthase), post-exercise blood lactate levels, skeletal muscle fiber types, or other markers of metabolic or cardiovascular adaptation (Holloszy and Coyle, 1984; Booth et al., 2010; Hellsten and Nyberg, 2015). If studies reported more than one outcome variable, performance outcomes based on the results of an exercise performance test were prioritized over other outcomes (Vesterinen et al., 2014). Studies that involved mice receiving a treatment other than exercise on a treadmill such as a diet or drug intervention were excluded. For studies that included four or more groups of mice – a control arm combined with exercise training (e.g., no treatment±exercise training) and a treatment arm combined with exercise training (treatment±exercise training), only the mice in the control arm were included in the analysis. Genetic manipulation or modification can have a significant impact on exercise performance. Therefore, this review focused on inbred or wild-type mice of any strain. Studies utilizing only transgenic or genetically manipulated mice were excluded as were mice performing swimming, wheel running, or other forms of exercise training. Several studies utilized a treadmill-based overtraining paradigm. Because this paradigm generally resulted in decreased performance, cohorts undergoing overtraining were excluded. However, if a traditional exercise training paradigm was included as part of the study and efficacy data available, data from mice in those cohorts were included. Any studies involving other animals or humans were excluded as were studies that did not report sufficient training efficacy data.

### Study Selection and Data Extraction

Following the initial search, titles and abstracts were screened for (1) inbred mice with no treatment; (2) inclusion of a sedentary/control group; (3) exercise training; and (4) training with a treadmill. Full-text articles were then assessed against the inclusion criteria. Data extracted included: author names, publication date and journal citation, sex and age of the mice, number of mice per group, exercise training protocol variables – frequency (days/week), session duration (min), treadmill velocity (m/min), treadmill incline (degrees), training duration (weeks), intensity (% of maximum), type of exercise performance test, and exercise training efficacy outcome variables for each group. In studies where the exercise protocol progressively increased to a maximal target workload, the final workload was used in all analyses. In some studies, the subject characteristics (e.g., age) or final training protocol variables (e.g., treadmill velocity) were presented as a range. In those cases, the median value was used for all analyses. The mean and standard deviation (SD) or standard error of the mean (SEM) were recorded for each outcome variable. If the outcome data were presented in figures, data were extracted using WebPlotDigitizer.^1^ Two investigators extracted data independently. A third investigator reviewed the data, calculated the average, or requested a re-analysis by both investigators.

### Quality Assessment of Included Studies

Risk of bias was assessed using a modified version of the CAMARADES checklist items (Macleod et al., 2004). The following reported items were recorded: (1) random assignment to groups, (2) blinded assessment of outcome variables, (3) sample size calculation, (4) animal welfare statement, and (5) conflict of interest statement.

### Data Analysis

All descriptive statistics were performed using JMP Pro 15 (SAS, Cary, NC, United States). Summary figures were generated using Prism 9 (GraphPad Software, La Jolla, CA, United States). All meta-analyses were conducted using Comprehensive Meta-Analysis Software v3 (Biostat Inc., Englewood, NJ). Statistical significance was set at *p*<0.05. Outcome variables were reported as pre- and post-training values, post-training only values, or as changes in the outcome variable. Standardized mean difference values between exercise-trained and sedentary groups (exercise group minus control group) were calculated as Hedges’ *g*. Positive values indicate an improvement with exercise training. If change score SD were not available, these were calculated using the study-specific correlation coefficient or a correlation coefficient of 0.6 between pre- and post-training values. The latter value is the mean of previously published correlation coefficients between pre- and post-training values for exercise performance phenotypes (Troxell et al., 2003; Massett and Berk, 2005; Avila et al., 2017). For studies with more than one exercise training group, the common control group was split into two groups with smaller samples sizes to avoid double counting of animals (Vesterinen et al., 2014). Standardized mean differences were calculated for each comparison and considered separate studies in all analyses. Thresholds were set as small, |*g*|≤0.5; medium, |*g*|<1.0; large, |*g*|<1.5; and very large, |*g*|≥1.5 (Labots et al., 2016).

To investigate the contribution of moderator variables on the effect of exercise training, study-level categorical and continuous variables were included individually and together in a random-effects meta-regression model. Categorical factors included strain, sex, exercise intensity, exercise performance test, and training outcome. Continuous variables included age, treadmill velocity and incline, frequency, time/session, and training duration.

Heterogeneity was evaluated using Cochran’s *Q* test, *I^2^*, and Tau^2^. Prediction intervals were calculated using CMA prediction interval program.^2^ Subgroup analysis was used to investigate the heterogeneity between the sample estimates based on study-level moderators: mouse strain, age, sex, outcome variable, exercise performance test type, and exercise training protocol variables.

To assess publication bias, the funnel plot of Hedges’ *g* vs. standard error, Egger’s regression, and Duval and Tweedie trim and fill were examined. Assuming a positive effect of exercise training on outcome variables, imputed missing studies were plotted to the left side of the mean.

## Results

### Selection Results

**Figure 1:**
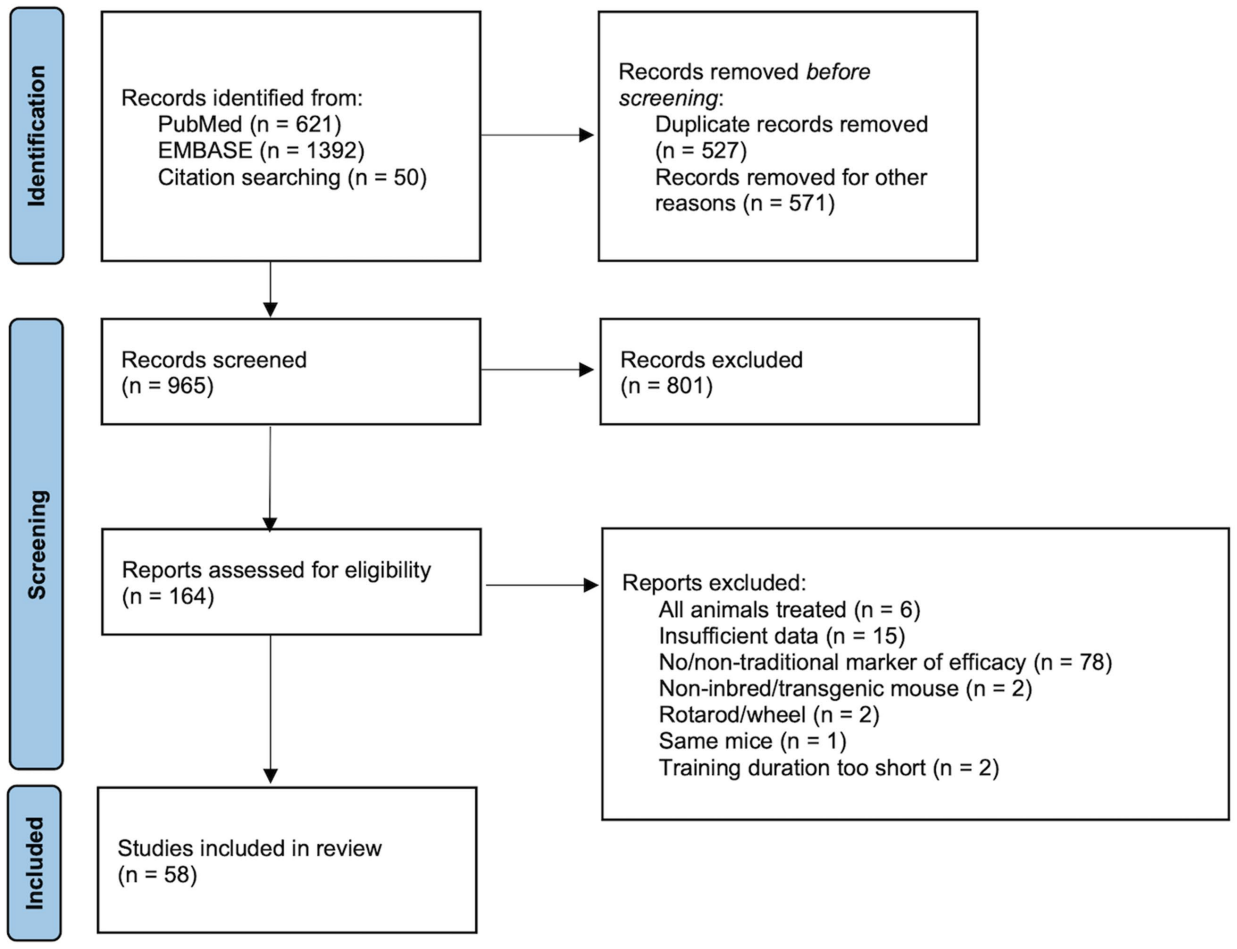
Flow chart of article selection process.

In the initial search, 2,063 articles were identified through database searches and other sources (i.e., reference lists and author publications). A flow chart based on PRISMA guidelines is shown in Figure 1 Page et al. (2021)). Duplicate records (*n*=527), non-full-text items (*n*=565), and non-English language items (*n*=6) were excluded. Of the remaining 965 articles, 801 articles were excluded based on the title and abstract review for: (1) inbred mice with no treatment, (2) inclusion of a sedentary/control group, (3) exercise training, and (4) training with a treadmill. The full text of 164 potentially eligible articles were assessed for inclusion criteria including markers of training responses. Of these, 106 articles were excluded for (1) utilizing transgenic/genetically modified mice, (2) including drug/diet supplement/treatment, (3) having different modalities of exercise such as running wheels and rotarod, or (4) no relevant exercise training phenotype. Fifty-eight (58) articles met the eligibility criteria and were included in the meta-analysis. Studies included in the systematic review and meta-analysis are summarized in Table 1.

**Table 1:** Summary of mouse characteristics and training parameters from studies included in meta-analysis.

Study	Subject Characteristics	Training Protocol	Outcome
Abadi et al., 2013	*Strain(s):* C57BL/6 J *Sex:* Female and Male *Age:*	*Frequency:* *Velocity:* 16–18 m/min *Incline:* *Session duration:* 45 min *Training duration:* 8 weeks *Intensity:*	Distance (m), Incremental load test
Aguiar et al., 2008	*Strain(s): CF*-1 *Sex:* Male *Age:* 6 weeks	*Frequency:* 5 d/wk. *Velocity:* 16.5 m/min *Incline:* 0° (Level group) *Session duration:* 45 min *Training duration:* 8 weeks *Intensity:*	Citrate synthase activity (mmol•min^−1^• g^−1^), soleus
Almeida-Oliveira et al., 2019	*Strain(s):* C57BL/6 *Sex:* Male *Age:* 8 weeks	*Frequency:* 5 d/wk. *Velocity:* *Incline:* *Session duration:* 60 min *Training duration:* 4 weeks *Intensity:* 50% maximal exercise capacity	Time (min), Incremental load test
Alves et al., 2017	*Strain(s):* C57BL/6 *Sex:* Male *Age:* 13 weeks	*Frequency:* 5 d/wk. *Velocity:* *Incline:* *Session duration:* 60 min *Training duration:* 7 weeks *Intensity:* 55–65% of maximal speed	Distance (m), Incremental load test
Alves et al., 2019	*Strain(s):* C57BL/6 *Sex:* Male *Age:* 13 weeks	*Frequency:* 5 d/wk. *Velocity:* *Incline:* *Session duration:* 60 min *Training duration:* 12 weeks *Intensity:* 70% maximal exercise capacity	Work (Joules), Incremental load test
Alves et al., 2020	*Strain(s):* C57BL/6 *Sex:* Male *Age:* 8 weeks	*Frequency:* 5 d/wk. *Velocity:* *Incline:* 14° *Session duration:* 60 min *Training duration:* 12 weeks *Intensity:* 60% of maximal speed	% change in velocity, Incremental load test
Avila et al., 2017	*Strain(s):* 24 strains *Sex:* Male *Age:* 8 weeks	*Frequency:* 5 d/wk. *Velocity:* 15–23 m/min *Incline:* 5–10° *Session duration:* 60 min *Training duration:* 4 weeks *Intensity:* 65%	Change in time (min), graded exercise test
Bartalucci et al., 2012	*Strain(s):* C57BL *Sex:* Male *Age:* 10 weeks	*Frequency:* 5 d/wk. *Velocity:* *Incline:* *Session duration:* *Training duration:* *Intensity:* HIT: 2 min @ 90% max, 1 min recovery to 1,000 meters, LOW: 60% of maximal velocity to 1,000 meters	Blood lactate concentration (mmol·L^−1^), at the end of the first and last training session
Boehnke et al., 1987	*Strain(s):* Swiss Webster *Sex:* Female *Age:* 9.5 weeks	*Frequency:* 6 d/wk. *Velocity:* moderate: 7 m/min, high: 15 m/min *Incline:* 6° *Session duration:* 60 min *Training duration:* 9 weeks *Intensity:*	Succinate dehydrogenase activity (umol/g tissue x min), gastrocnemius
Borg et al., 2014	*Strain(s):* C57BL/6 J *Sex:* Male *Age:* 12 weeks	*Frequency:* 5 d/wk. *Velocity:* 18 m/min *Incline:* 5° *Session duration:* 70 min *Training duration:* 6 weeks *Intensity:*	Time (s), Incremental load test
De Angelis et al., 2004	*Strain(s):* C57/6 J *Sex:* Male *Age:*	*Frequency:*5 d/wk. *Velocity:* 17 m/min *Incline:* *Session duration:* 60 min *Training duration:* 4 weeks *Intensity:* 50–70% maximal running speed	Speed (km/h), graded exercise test
Durigan et al., 2009a	*Strain(s):* BALB/c *Sex:* Male *Age:* 16 weeks	*Frequency:* 5 d/wk. *Velocity:* *Incline:* *Session duration:* 60 min *Training duration:* 12 weeks *Intensity:* 50% maximal speed and 75% maximal speed	Improvement in maximal exercise capacity (min), incremental load test
Durigan et al., 2009b	*Strain(s):* BALB/c *Sex:* Male *Age:* 16 weeks	*Frequency:* 5 d/wk. *Velocity:* *Incline:* *Session duration:* 60 min *Training duration:* 12 weeks *Intensity:* 50% maximal speed and 75% maximal speed	Time (min), Incremental load test
Ferreira et al., 2007	*Strain(s):* C57BL/6 J *Sex:* Male *Age:* 20 weeks	*Frequency:* 5 d/wk. *Velocity:* 15.1 m/min *Incline:* *Session duration:* 60 min *Training duration:* 8 weeks *Intensity:* MLSSw	Total distance run (m), Incremental load test
Fiuza-Luces et al., 2018	*Strain(s):* C57BL/6 J *Sex:* Male *Age:* 8 weeks	*Frequency:* 5 d/wk. *Velocity:* *Incline:* 8.5° *Session duration:* 50 min *Training duration:* 8 weeks *Intensity:* 70–75% Vmax	Total running distance (m), Incremental load test
Foryst-Ludwig et al., 2011	*Strain(s):* C57BL/6 J *Sex:* Female, Male *Age:* 5 weeks	*Frequency:* 7 d/wk. *Velocity:* 15 m/min *Incline:* 7° *Session duration:* 90 min *Training duration:* 3 weeks progressive increase and 4 weeks at final workload *Intensity:*	Blood lactate (mg/dl)
German and Hoffman-Goetz, 1986	*Strain(s):* C57BL/6 J *Sex:* Male *Age:* 72–80 weeks	*Frequency:* 5 d/wk. *Velocity:* 15 m/min *Incline:* 0° *Session duration:* 30 m/min *Training duration:* 8 weeks *Intensity:*	Succinate dehydrogenase activity (μmoles/gm protein/min), vastus intermedius
Han, 2013	*Strain(s):* 129 SvJ/C57BL6 *Sex:* Male *Age:* 15 weeks	*Frequency:* 5 d/wk. *Velocity:* 24 m/min *Incline:* *Session duration:* 40 min *Training duration:* 8 weeks *Intensity:*	Ratio of heart weight (mg) to body weight (g)
Herbst et al., 2015	*Strain(s):* C57BL/6 *Sex:* Male *Age:* 7 weeks	*Frequency:* 5 d/wk. *Velocity:* 25 m/min *Incline:* 11.3° *Session duration:* 60 min *Training duration:* 4 weeks *Intensity:*	mtDNA copy number (vastus lateralis)
Hoffman-Goetz et al., 1986	*Strain(s):* C57BL/6 J *Sex:* Male *Age:* 12 weeks	*Frequency:* 5 d/wk. *Velocity:* 28 m/min *Incline:* 8° *Session duration:* 30 min *Training duration:* 4 weeks *Intensity:*	Succinate dehydrogenase activity (μmoles/g protein/min), quadriceps femoris
Hoffman-Goetz et al., 1989	*Strain(s):* C3He *Sex:* Male *Age:* 8 weeks	*Frequency:* 5 d/wk. *Velocity:* 30 m/min *Incline:* 8° *Session duration:* 30 min *Training duration:* 8 weeks *Intensity:*	Succinate dehydrogenase activity (μmoles/g protein/min), quadriceps femoris
Ingalls et al., 1996	*Strain(s):* ICR *Sex:* Male *Age:* 7 weeks	*Frequency:* 4 d/wk. *Velocity:* 27–36 m/min *Incline:* 9.9° *Session duration:* 3 sets of 3 min (30 s recovery) *Training duration:* 8 weeks *Intensity:*	Work output (joules), Incremental load test
Jadeski and Hoffman-Goetz, 1996	*Strain(s):* C3H/HeJ *Sex:* *Age:* 4–9 weeks	*Frequency:* 5 d/wk. *Velocity:* 20 m/min *Incline:* 0° *Session duration:* 30 min *Training duration:* 9 weeks *Intensity:*	Citrate synthase activity (mol/min/g tissue), soleus
Kaurstad et al., 2012	*Strain(s):* C57BL/6 J *Sex:* Female *Age:* 8 weeks	*Frequency:* 5 d/wk. *Velocity:* *Incline:* 25° *Session duration:* 60 min *Training duration:* 6 weeks *Intensity:* 4 min @ 85–90% $\dot{V}$ O_2max_, 2 min @ 50–60% $\dot{V}$ O_2max_	Maximal oxygen uptake ( $\dot{V}$ O_2max_) (ml/kg^0.75^/min)
Kemi et al., 2002	*Strain(s):* C57BL/6 J *Sex:* Female and Male *Age:* 7–8 weeks	*Frequency:* 5 d/wk. *Velocity:* *Incline:* 25° *Session duration:* 120 min *Training duration:* 8 weeks *Intensity:* 8 min @ 85–90% $\dot{V}$ O_2max_, 2 min @ 50–60% $\dot{V}$ O_2max_	Maximal oxygen uptake ( $\dot{V}$ O_2max_) (ml/kg^0.75^/min)
Kim et al., 2019	*Strain(s):* ICR *Sex:* Male *Age:* 7 weeks	*Frequency:* 5 d/wk. *Velocity:* 18 m/min *Incline:* 8° *Session duration:* 50 min *Training duration:* 4 weeks *Intensity:* 60% $\dot{V}$ O_2max_	Oxygen uptake during 1 h of exercise (ml/kg/min)
Kim et al., 2020	*Strain(s):* C57BL/6 J, 129S1/SvImJ, SJL/J, NON/ShiLtJ *Sex:* Male *Age:* 8 weeks	*Frequency:* 5 d/wk. *Velocity:* *Incline:* *Session duration:* HIT: 60 min, MOD: 70 min *Training duration:* 4 weeks *Intensity:* HIT: 8 min @ 85% max speed, 2 min @ 50% max speed; MOD: 65% maximal speed	Change in time (min), Graded exercise test
Kruger et al., 2013	*Strain(s):* C57BL/6 N *Sex:* Male *Age:* 10–12 weeks	*Frequency:* 5 d/wk. *Velocity:* 12 m/min *Incline:* *Session duration:* 35 min *Training duration:* 10 weeks *Intensity:*	Maximal oxygen consumption ( $\dot{V}$ O_2max_) (ml/min/kg)
Kruger et al., 2016	*Strain(s):* C57BL/6 N *Sex:* Male *Age:* 10–12 weeks	*Frequency:* 5 d/wk. *Velocity:* 15.6 m/min *Incline:* *Session duration:* 35 min *Training duration:* 10 weeks *Intensity:* 80% $\dot{V}$ O_2max_	Maximal oxygen consumption ( $\dot{V}$ O_2max_) (ml/min/kg)
Lee et al., 2015	*Strain(s):* C57BL/6 N *Sex:* Male *Age:* 56 weeks	*Frequency:* 5 d/wk. *Velocity:* *Incline:* 5° *Session duration:* 60 min *Training duration:* 8 weeks *Intensity:* 60% of maximum work rate	Total distance run (m), Incremental load test
Lehti et al., 2006	*Strain(s):* NMRI *Sex:* Male *Age:* 10 weeks	*Frequency:* 5 d/wk. *Velocity:* 21 m/min *Incline:* 2.5° *Session duration:* 60 min *Training duration:* 5 weeks *Intensity:*	Citrate synthase activity (nmol•min^−1^ •mg^−1^), calf muscle complex
Liu et al., 2008	*Strain(s):* BALB/c *Sex:* Male *Age:* 12 weeks	*Frequency:* 5 d/wk. *Velocity:* 10 m/min *Incline:* *Session duration:* 60 min *Training duration:* 4 weeks *Intensity:* moderate	Citrate synthase activity (μmol/min/mg protein), soleus
Lucchetti et al., 2017	*Strain(s):* Swiss *Sex:* Male *Age:*	*Frequency:* 5 d/wk. *Velocity:* 15.1 m/min *Incline:* *Session duration:* 60 min *Training duration:* 9 weeks *Intensity:*	Maximum speed (m/min), Incremental load test
Malek et al., 2013	*Strain(s):* FVB/NJ *Sex:* Male *Age:* 16 weeks	*Frequency:* 5 d/wk. *Velocity:* *Incline:* 5° *Session duration:* 30 min continuous or 3 × 10 min (2 h recovery between) *Training duration:* 8 weeks *Intensity:* 60% of the maximal work rate	Time (seconds), Incremental load test
Massett and Berk, 2005	*Strain(s):* BALB/cJ, C57BL/6 J, FVB/NJ *Sex:* Male *Age:* 8 weeks	*Frequency:* 5 d/wk. *Velocity:* B6, BALB: 15 m/min, FVB: 19 m/min *Incline:* B6, BALB: 5°, FVB: 10° *Session duration:* 60 min *Training duration:* 4 weeks *Intensity:* ~60% of the maximal workload	Time (min), Graded exercise test
Meier et al., 2013	*Strain(s):* C57BL/6 *Sex:* Male *Age:* 9 weeks	*Frequency:* 5 d/wk. *Velocity:* 26 m/min *Incline:* 10° *Session duration:* 45 min *Training duration:* 4 weeks *Intensity:*	Time (seconds), Incremental load test
Mikami et al., 2004	*Strain(s):* ICR *Sex:* Male *Age:* 10 weeks	*Frequency:* 5 d/wk. *Velocity:* 25 m/min *Incline:* 0° *Session duration:* 60 min *Training duration:* 4 weeks *Intensity:*	Citrate synthase activity (U/mg protein), soleus
Niebauer et al., 1999	*Strain(s):* C57BL/6 J *Sex:* Female *Age:* 8 weeks	*Frequency:* 6 d/wk. *Velocity:* 22 m/min *Incline:* 8° *Session duration:* 120 min (2 × 1 h/day) *Training duration:* 4 weeks *Intensity:* 85% of maximal oxygen uptake	Maximal oxygen consumption ( $\dot{V}$ O_2max_) (ml/min/kg)
Niel et al., 2017	*Strain(s):* C57BL/6 *Sex:* Male *Age:* 92 weeks	*Frequency:* *Group 1*: 5 d/wk., *Group 2*: 5 sessions over 2 weeks *Velocity:* *Group 1*: 14 m/min, *Group 2*: 3 m.min^−2^ × 11 min, 6 m.min^−2^ × 6 min, 12 m.min^−2^ × 3 min (30 min rest between) *Incline:* 0° *Session duration: Group 1*: 60 min continuous, *Group 2*: 20 min *Training duration: Group 1*: 4 weeks, *Group 2*: 2 weeks *Intensity:* 50% of the maximum running speed (Vpeak)	Time (min), Incremental load test
Pereira et al., 2012	*Strain(s):* Swiss *Sex:* Male *Age:* 8 weeks	*Frequency:* 5 d/wk. *Velocity:* *Incline:* 0° *Session duration:* 60 min *Training duration:* Trained: 8 weeks, Overtrained: 4 weeks (before overtraining protocol) *Intensity:* 60% of exhaustion velocity	Exhaustion time (min), Incremental load test
Pereira et al., 2013	*Strain(s):* Swiss *Sex:* Male *Age:* 8 weeks	*Frequency:* 5 d/wk. *Velocity:* *Incline:* 0° *Session duration:* 60 min *Training duration:* Trained: 8 weeks, Overtrained: 4 weeks (before overtraining protocol) *Intensity:* 60% of exhaustion velocity	Exhaustion time (min), Incremental load test
Pereira et al., 2014a	*Strain(s):* Swiss *Sex:* Male *Age:* 8 weeks	*Frequency:* 5 d/wk. *Velocity:* *Incline:* 0° *Session duration:* 60 min *Training duration:* 8 weeks *Intensity:*	Percentage change between week 0 and week 8 for time to exhaustion, Incremental load test
Pereira et al., 2014b	*Strain(s):* Swiss *Sex:* Male *Age:* 8 weeks	*Frequency:* 5 d/wk. *Velocity:* *Incline:* 0° *Session duration:* 60 min *Training duration:* 8 weeks *Intensity:*	Percentage change between week 0 and week 8 for exhaustion velocity, Incremental load test
Pereira et al., 2015	*Strain(s):* C57BL/6 *Sex:* Male *Age:* 8 weeks	*Frequency:* 5 d/wk. *Velocity:* *Incline:* 0° *Session duration:* 60 min *Training duration:* 4 weeks (before start of overtraining protocol) *Intensity:* 60% of exhaustion velocity	Exhaustion velocity (m/min), Incremental load test
Pereira et al., 2016	*Strain(s):* C57BL/6 *Sex:* Male *Age:*	*Frequency:* 5 d/wk. *Velocity:* *Incline:* *Session duration:* 60 min *Training duration:* 4 weeks *Intensity:* 60% of maximal velocity	Maximal velocity (km/h), Incremental load test
Pinto et al., 2015	*Strain(s):* C57BL/6 N *Sex:* Male *Age:* 12 weeks	*Frequency:* 5 d/wk. *Velocity:* 15 m/min *Incline:* *Session duration:* 60 min *Training duration:* 6 weeks *Intensity:*	Systolic blood pressure (tail-cuff)
Rodrigues et al., 2019	*Strain(s):* C57BL/6 J *Sex:* Male *Age:* 20 weeks	*Frequency:* 5 d/wk. *Velocity:* *Incline:* *Session duration:* 60 min *Training duration:* 8 weeks *Intensity:* 60% of maximal speed	Total distance run (m), Incremental load test
Savage and McPherron, 2010	*Strain(s):* C57BL/6 *Sex:* Male *Age:* 12 weeks	*Frequency:* 5 d/wk. *Velocity:* 12 m/min *Incline:* 0° *Session duration:* 30 min *Training duration:* 4 weeks *Intensity:*	Citrate synthase activity (mu/μg protein), quadriceps
Sousa et al., 2019	*Strain(s):* C57BL6/JUnib *Sex:* Male *Age:* 6–7 weeks	*Frequency:* 5 d/wk. *Velocity:* *Incline:* 0° *Session duration:* 60 min *Training duration:* 8 weeks *Intensity:* 10 min at 40% of maximal speed, 40 min at 50–60% of maximal speed, and 10 min at 40% of maximal speed	Time (min), Incremental load test
Steiner et al., 2011	*Strain(s):* ICR *Sex:* Male *Age:* 8 weeks	*Frequency:* 6 d/wk. *Velocity:* 25 m/min *Incline:* 2.9° *Session duration:* 60 min *Training duration:* 8 weeks *Intensity:*	Time (min), Run to fatigue test
Sturgeon et al., 2015	*Strain(s):* C57BL/6 J *Sex:* Female *Age:* 8 weeks	*Frequency:* 5 d/wk. *Velocity:* 18 m/min *Incline:* *Session duration:* 60 min *Training duration:* 8 weeks *Intensity:*	Work (m•kg), Incremental load test
Suominen et al., 1980	*Strain(s):* NMRI *Sex:* Male *Age:* 3 weeks and 8 weeks	*Frequency:* 5 d/wk. *Velocity:* 18 m/min *Incline:* 5° *Session duration:* 80 min (2 × 40 min) *Training duration:* 4 weeks (3 wo mice), 3 weeks (8 wo mice) *Intensity:*	Dry weight of heart. (mg)
Toti et al., 2013	*Strain(s):* C57BL *Sex:* Male *Age:* 10 weeks	*Frequency:* 5 d/wk. *Velocity:* LOW: 17.1 m/min, HIT: 33.75 m/min *Incline:* *Session duration:* time to complete running 1,000 meters *Training duration:* 8 weeks *Intensity:* HIT: 90% of maximal running velocity for 2 min, 1 min recovery; LOW: 60% of maximal running velocity	Blood lactate concentrations (mmol•L^−1^) before 1st training session and after last training session
Uddin et al., 2016	*Strain(s):* C57BL/6 J *Sex:* Female *Age:* 11 weeks at start of training	*Frequency:* 6 d/wk. *Velocity:* 15 m/min *Incline:* *Session duration:* 45 min *Training duration:* 6 weeks *Intensity:*	Citrate synthase activity (μmol/min/mg protein), quadriceps
Vieira et al., 2008	*Strain(s):* BALB/c *Sex:* Male *Age:*	*Frequency:* 5 d/wk. *Velocity:* *Incline:* *Session duration:* 60 min *Training duration:* 4 weeks *Intensity:* Low: 50% of maximal speed, Moderate: 75% of maximal speed	Time (min), Incremental load test
Vihko et al., 1979	*Strain(s):* NMRI *Sex:* Male *Age:* 9–11 weeks	*Frequency:* 5 d/wk. *Velocity:* 25 m/min *Incline:* 6° *Session duration:* 90 min *Training duration:* *Intensity:*	Citrate synthase activity (nmol substrate consumed/min per mg muscle), red Musculi quadriceps femoris (MQF)
Wernig et al., 1991	*Strain(s):* CBA/J *Sex:* Male *Age:* 12 weeks	*Frequency:* *Velocity:* 14 m/min *Incline:* 6° *Session duration:* 540 min (3 × 3 h with 30 min rest in the cages between the bouts) *Training duration:* 8–10 times at intervals of 3–5 days *Intensity:*	Total number of Type II fibers, soleus
Woods et al., 2003	*Strain(s):* BALB/c *Sex:* Male *Age:* 8 weeks and 72 weeks	*Frequency:* 5 d/wk. *Velocity:* 13–22 m/min *Incline:* *Session duration:* 45 min *Training duration:* 16 weeks *Intensity:*	Citrate synthase activity (μm g wet wt^−1^ min^−1^), soleus

### Quality of the References and Publication Bias

**Figure 2:**
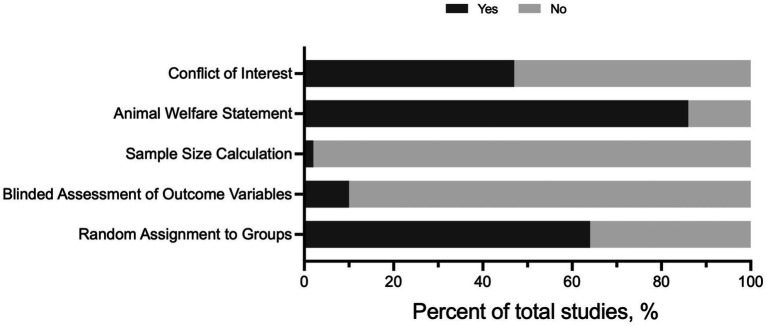
Risk of bias assessment for 58 full-text articles meeting inclusion criteria.

Publication study quality was assessed in the full-text articles included for review (Figure 2). Out of 58 full-text articles, less than ~2% of the articles included a sample size calculation. Moreover, only six full-text articles (10%) included blinded assessment of the outcome. In general, the blinding assessment was applied to outcome variables not relevant to traditional exercise training-related phenotypes and not to group assignment or exercise performance tests. Random assignment to sedentary (SED) and exercise training (EX) groups was documented in 38 articles; however, the methods for randomization were not provided. The majority of the articles included an animal welfare statement (86%, 50/58 articles) and a conflict-of-interest statement (47%, 27/58). Collectively, these findings suggest there may be a potential bias in published articles, especially regarding appropriate sample size and blinding of outcomes.

**Figure 3:**
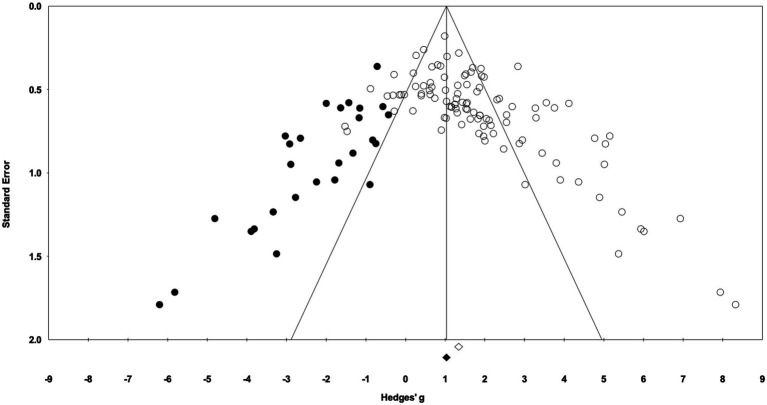
Funnel plot of Hedges’ *g* between exercise training and sedentary control groups. Open diamond indicates the point estimate and 95% CI for the combined studies using a random effects model. The black diamond indicates the point estimate based on the Duval and Tweedie’s Trim and Fill analysis using a random effects model. Black circles are imputed studies from Trim and Fill analysis.

In the 58 articles meeting the eligibility criteria, several reported results for more than one exercise training – sedentary cohort (e.g., multiple strains or sexes). For data analysis, each distinct training group (i.e., EX-SED pair) was considered a separate study, therefore, data from 105 studies are reported (i.e., 105 EX-SED pair comparisons). The assumption prior to starting this review was that there would be a significant bias toward the beneficial effects of exercise training; therefore, several approaches were used to assess publication bias. A significant effect of exercise training was observed in approximately 70% of included studies. The funnel plot in Figure 3 shows the distribution of studies. A greater number of studies are located to the right of the mean effect size, suggesting some degree of publication bias. The random effects model point estimate and 95% CI for the combined studies was 1.70 (95% CI: 1.47–1.94). Using Trim and Fill the point estimate was 1.08 (95% CI: 0.82–1.35) with approximately 27 missing studies (Figure 3). The asymmetry was confirmed by the Egger’s test. The intercept of the regression was 3.11 (95% CI: 2.11–4.12), with *t*=6.15, df=103, one-tailed value of *p*<0.05. The result from the Egger’s Test indicates significant asymmetry in the funnel plot (Egger et al., 1997).

### Subject Characteristics

Data from 2,049 mice were reported in the 105 included studies. Twenty-eight different mouse strains were used in 58 full-text articles. C57BL/6 was the most used strain (39%), followed by BALB/c (10.5%), Swiss (9.5%), and NMRI, ICR, and FVB/NJ (3.8% each) strains. There was a marked difference in the number of studies that used male or female mice. Male mice were used exclusively in 88% of studies, whereas only a few studies (9%) utilized female mice. Three studies included both male and female mice (Kemi et al., 2002; Foryst-Ludwig et al., 2011; Abadi et al., 2013). The median age of mice was 8weeks old with a range of 3–92weeks old (mean±SD, 13±16weeks) suggesting that most studies were conducted using younger adult mice. Seven percent of studies did not report the age of the mice. On average, sedentary control and exercise training groups included 9±9 (mean±SD) mice and 10±8 mice per study, respectively.

### Training Protocols

There was a wide range of treadmill training protocols reported. Most studies included information about the training protocol components: frequency of exercise (days/week), velocity of the treadmill, incline of the treadmill, length of each session (time in minutes), and the duration of the exercise training (weeks). Treadmill velocity was reported as m/min, m/s, or cm/s. Treadmill incline was reported in degrees or % incline. Velocity and incline were converted to m/min and degrees for data analysis. The mode for each parameter was: frequency of 5days/week (91% of studies, range: 2–7days/week), a treadmill velocity of 15m/min (19%, 5.25–33.8m/min), 10° of treadmill incline (38%, 0–25°), 60min/session (64%, 9–540min/session), and a duration of 4weeks (53%, 2–16weeks). Fifty studies (48%) included information for all components of the training protocol. The number of studies with missing exercise protocol data was: frequency: *n*=3missing, treadmill velocity: *n*=38, treadmill incline: *n*=39, time per session: *n*=4, and training duration: *n*=2. Studies not reporting some or all these components typically listed exercise intensity instead. Exercise intensities were reported as low, moderate, high or as a percentage of maximum.

### Exercise Tests

Performance outcomes (i.e., time, work, or distance) based on the results of an exercise performance test were reported in 78 studies. Exercise testing was not uniform in these studies and therefore was categorized by the testing protocol or outcome. Most testing protocols fell into three primary categories: Graded Exercise Testing (GXT), Incremental Load Testing (ILT), and $\dot{V}$O_2max_. A GXT that included incremental increases in both treadmill velocity and incline was utilized in 46% of studies reporting performance-based outcomes. ILT, an incremental increase in treadmill velocity and no change in incline, was reported in 46% of studies, and a $\dot{V}$O_2max_ protocol, measuring maximal oxygen consumption during exercise, was used in 8% of studies reporting performance-based outcomes. Submaximal endurance tests at a constant workload were used in a few other studies (Steiner et al., 2011; Kim et al., 2019).

### Outcomes

Most studies (74%) used a measure of exercise performance as a marker of exercise training efficacy (Table 1). Performance was measured during an exercise test (above) and reported as time, distance, maximal speed/velocity, or work. Differences in these outcome measures were compared between sedentary and exercise training groups. Twenty studies (19%) assessed training efficacy using biochemical measures including citrate synthase or succinate dehydrogenase enzyme activity in skeletal muscle, blood lactate concentrations, or mitochondrial DNA copy number (Table 1). Other outcome measures used were heart weight or heart weight to body weight ratios (Suominen et al., 1980; Foryst-Ludwig et al., 2011; Han, 2013), the number of type 2 skeletal muscle fibers (Wernig et al., 1991), and the systolic blood pressure before and after training (Pinto et al., 2015).

### Meta-Analysis

#### Overall Effect Size and Heterogeneity

**Figure 4:**
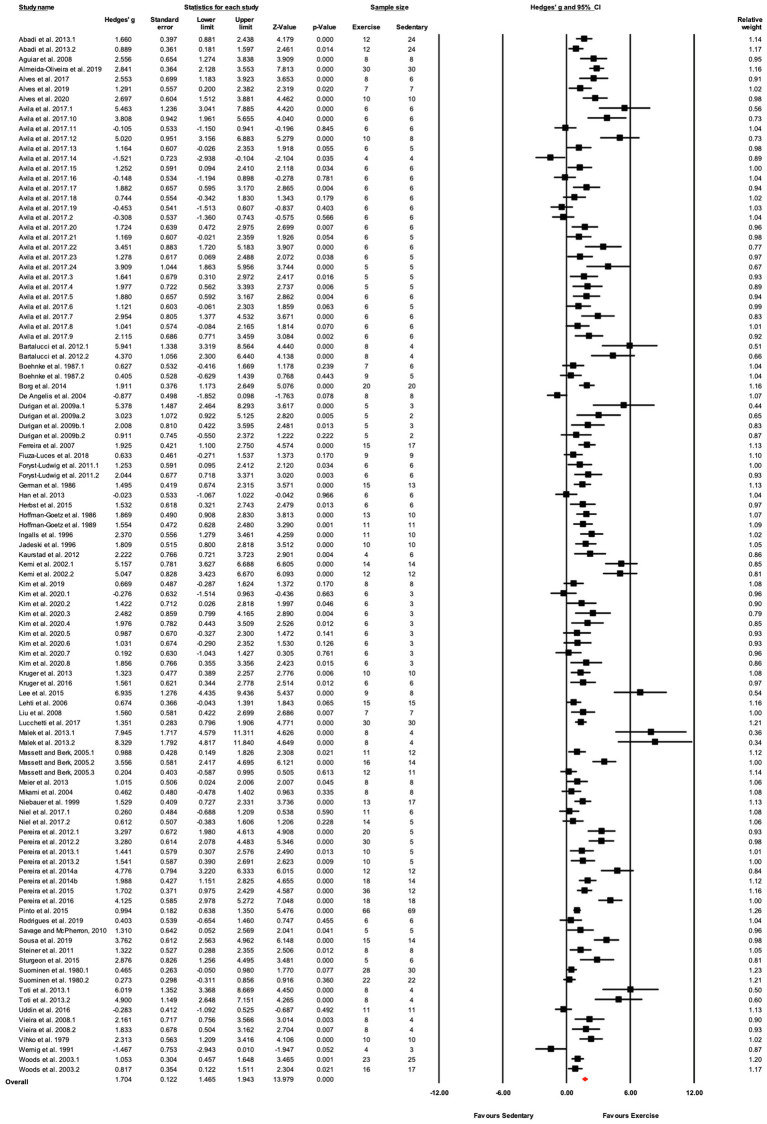
ContinuedFIGURE 4Mean difference effects of treadmill endurance training compared with sedentary control on markers of training efficacy. Standardized mean differences were calculated as Hedges’ *g*. Overall analysis was conducted using a random effects model. Values to the left of zero (Favors Sedentary) indicates the sedentary group had a greater response. Values to the right of zero (Favors Exercise) indicates a greater response in the exercise training group. The size of the black squares indicates the weight of the study-specific estimates. Red diamond indicates pooled estimate of random effects model.

The data from 105 studies was aggregated in the random effect model for the meta-analysis (Figure 4). The overall effect of exercise was statistically significant, with high heterogeneity (Hedges’s *g*=1.70, 95% CI=1.47–1.94, *p*<0.05, Tau^2^=1.14, *I^2^*=80.4%, prediction interval=−0.43–3.84). To investigate the heterogeneity across studies, subgroup analysis was performed using 10 moderator variables: strain, age, sex, training intensity, velocity, incline, time/session, duration, performance test, and the type of outcome variable (e.g., performance-based, biochemical, etc.). Performance tests included GXT, ILT, maximal oxygen consumption test ($\dot{V}$O_2max_). Table 2 shows the outcome of the subgroup analysis for each of the moderators. Treadmill incline, training duration, exercise performance test protocol, and outcome variable showed significant differences between subgroups. We also performed meta-regression to determine the percentage of heterogeneity explained by each moderator subgroup and by the combination of moderators related to the exercise training protocol. The results for the individual moderators are shown in Table 2. Five moderators, treadmill velocity, treadmill incline, exercise session time, performance test, and outcome variable category, each showed significant associations between moderator value and exercise training response. When training frequency, treadmill velocity and incline, time per exercise session, and training duration were included in the meta-regression as continuous variables, this model accounted for 0% of the between-study variance, suggesting that other factors are also contributing to differences between studies.

**Table 2:** Subgroup analyses for the effect of exercise training on markers of training efficacy in mice.

Moderator variable	Subgroups	Number of studies	Between-group differences			Meta-regression
			*Q*-value	df	*p*	*R*^2^ (%)
Strain	B6	41	2.77	1	ns	0
	Other	64				
Age	≤8weeks	66	0.22	1	ns	0
	>8weeks	32				
Sex	Male	95	0.0006	1	ns	0
	Female	9				
Training intensity	High	14	0.63	1	ns	0
	Moderate	58				
Treadmill velocity	≤10m/min	3	3.97	2	ns	3
	11–20m/min	43				
	>20m/min	21				
Treadmill incline	≤5°	24	6.36	2	<0.05	2
	6–10°	37				
	>10°	5				
Time/session	≤30min	11	2.40	3	ns	0
	31–45min	10				
	46–60min	66				
	>60min	14				
Training duration	≤4weeks	57	15.06	2	<0.05	0
	5–8weeks	32				
	>8weeks	14				
Performance test	GXT	36	11.34	2	<0.05	9
	ILT	36				
	$\dot{V}$O_2_	6				
Outcome variable	Biochemical	20	17.58	2	<0.05	3
	Performance	78				
	Other	7				

*GXT, graded exercise test; ILT, incremental load test; $\dot{V}$O_2max_, maximal oxygen consumption test; df, degrees of freedom; ns, non-significant *p*>0.05; *R*^2^ (%), regression coefficient*.

Based on the significant difference observed for subgroups of outcome variables (e.g., performance-based vs. biochemical; Table 2), separate meta-analyses were performed for studies with performance-based outcome variables and studies which reported biochemical-related outcome variables. There were too few studies coded as “Other” to support a separate analysis of studies in that category. Thus, two separate meta-analyses were done on two different groups of studies: (1) a group of studies with performance-based outcome variables, and (2) another group of studies that reported biochemical-related outcome variables.

##### Results for Performance-Based Outcome

**Figure 5:**
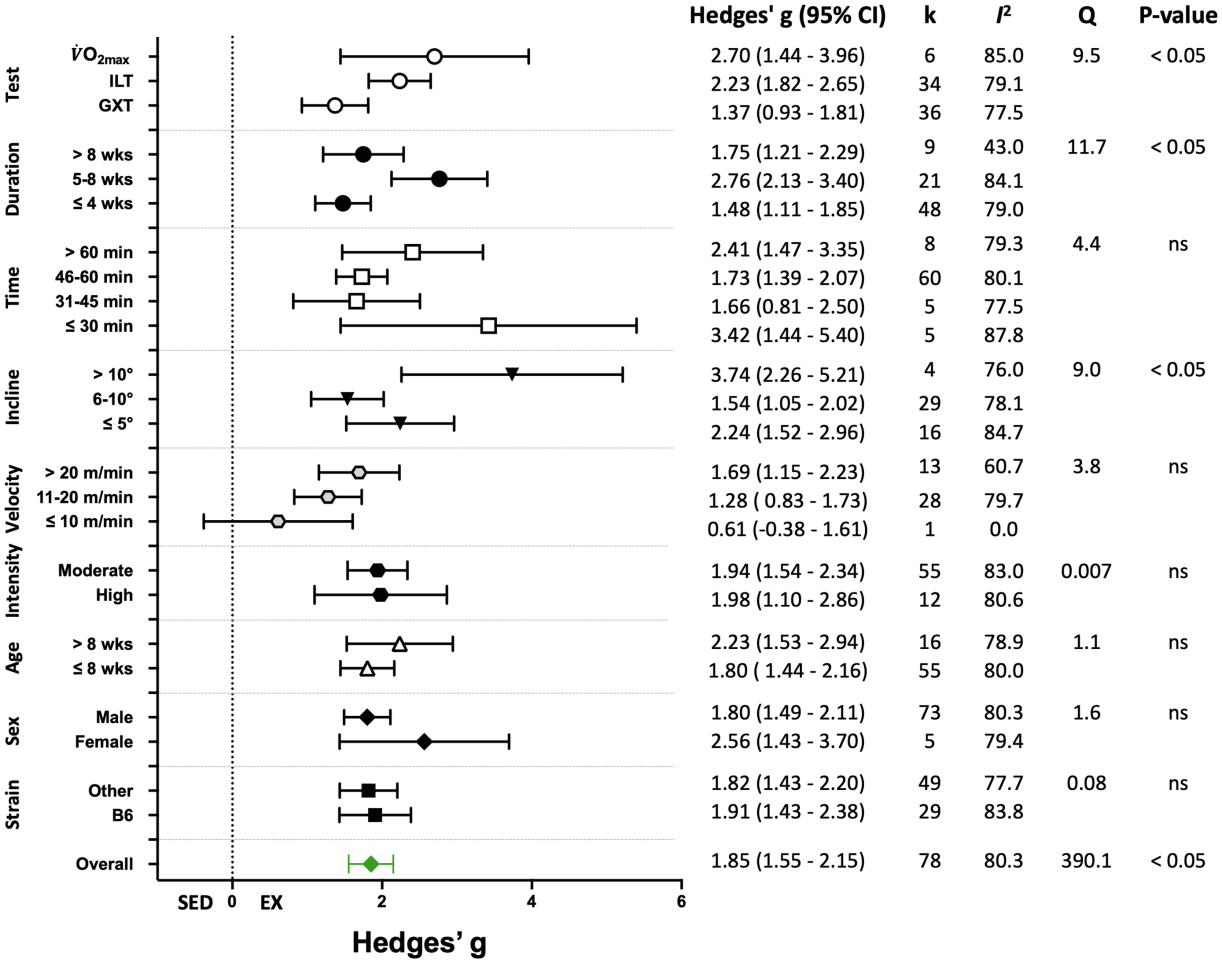
Adjusted effect sizes of the between-group comparisons for the predefined moderators on performance-based exercise training outcomes. B6, C57BL/6 mice; GXT, graded exercise test; ILT, incremental load test; $\dot{V}$O_2max,_ maximal oxygen consumption test; k, number of studies in each subgroup; *I*^2^, measure of heterogeneity; *Q*, Cochran’s *Q*; *p*, value of *p* for heterogeneity analysis (overall) or differences between subgroups; ns, non-significant *p*>0.05.

Seventy-eight (78) studies out of 105 (74%) included in the meta-analysis assessed performance-based outcome variables such as exhaustion time, maximum velocity, or work. The overall effect of exercise training on performance-based outcome variables from those studies was significant, with high between-study heterogeneity (Hedges’ *g*=1.85, 95% CI=1.55–2.15, *p*<0.05, *Q*-value=390.13, df=77, Tau^2^=1.35, *I^2^*=80.3%, prediction interval=−0.48 to 4.18). A summary of the subgroup analysis performed to investigate the heterogeneity across the studies reporting performance-based outcome variables is shown in Figure 5. Significant differences between subgroups were observed for treadmill incline, training duration, and the type of exercise test. Non-significant results were obtained from the subgroup analyses of strain, age, sex, exercise intensity, treadmill velocity, and time/session. Results for moderator variables with significant differences between subgroups are described below.

##### Grouped by Treadmill Incline

**Figure 6:**
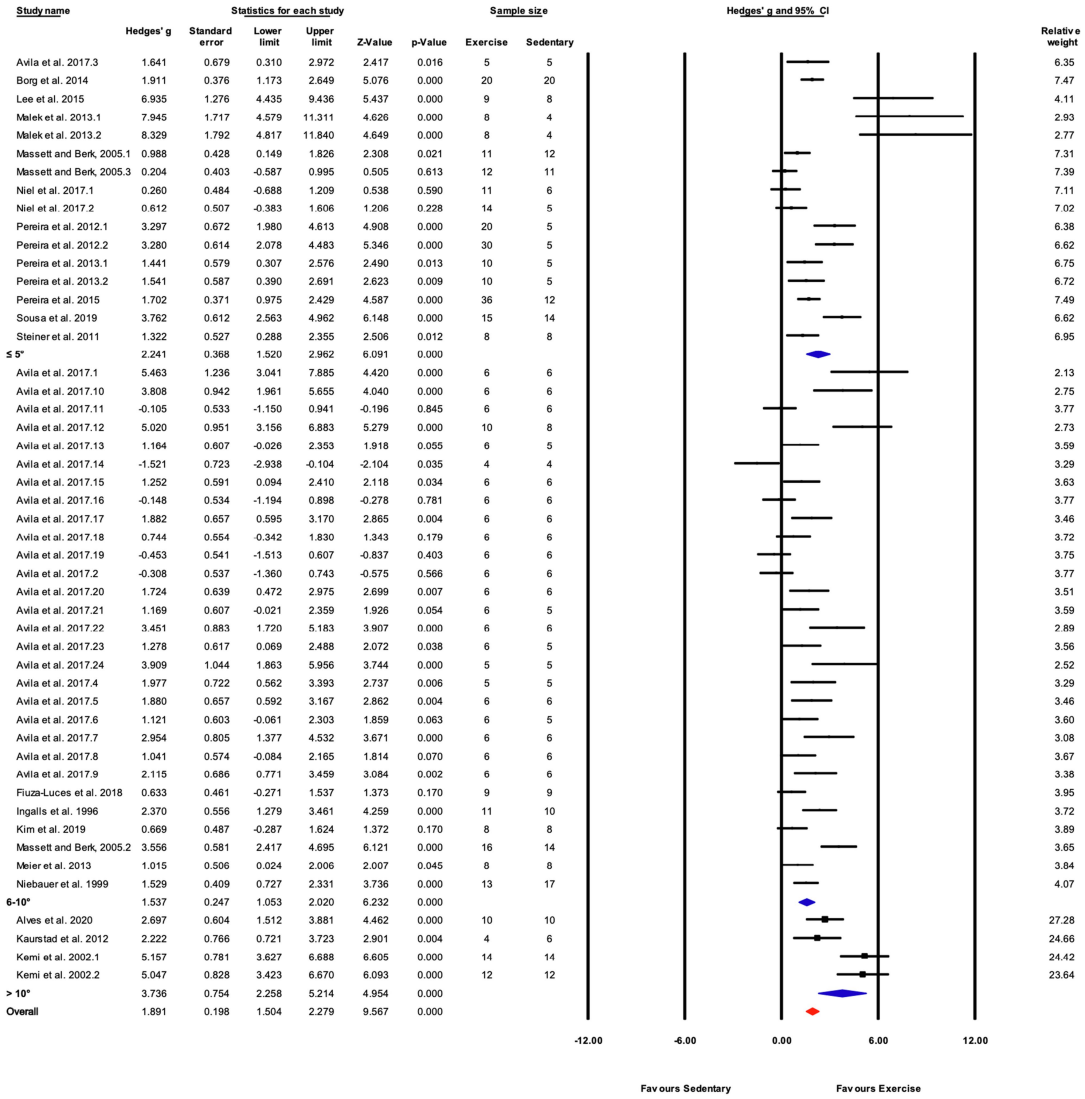
Continued FIGURE 6Forest plot of the between-group comparisons of the effect of training protocol duration on performance-based markers of training efficacy. Standardized mean differences were calculated as Hedges’ *g*. Overall analysis was conducted using a random effects model. Values to the left of zero (Favors Sedentary) indicates the sedentary group had a greater response. Values to the right of zero (Favors Exercise) indicates a greater response in the exercise training group. The size of the black squares indicates the weight of the study-specific estimates. Blue diamond indicates pooled estimate of random effects model for each subgroup. Red diamond indicates overall pooled estimate of random effects model.

When studies were divided based on treadmill incline, significant differences between trained and sedentary groups were observed regardless of the incline (Figures 5, 6). Studies that incorporated an incline >10° had a greater response to training relative to those with inclinations of ≤5° and 6–10° (*Q*-between=8.96, df=2, *p*<0.05, *I^2^*=82.6%).

##### Grouped by Training Duration

**Figure 7:**
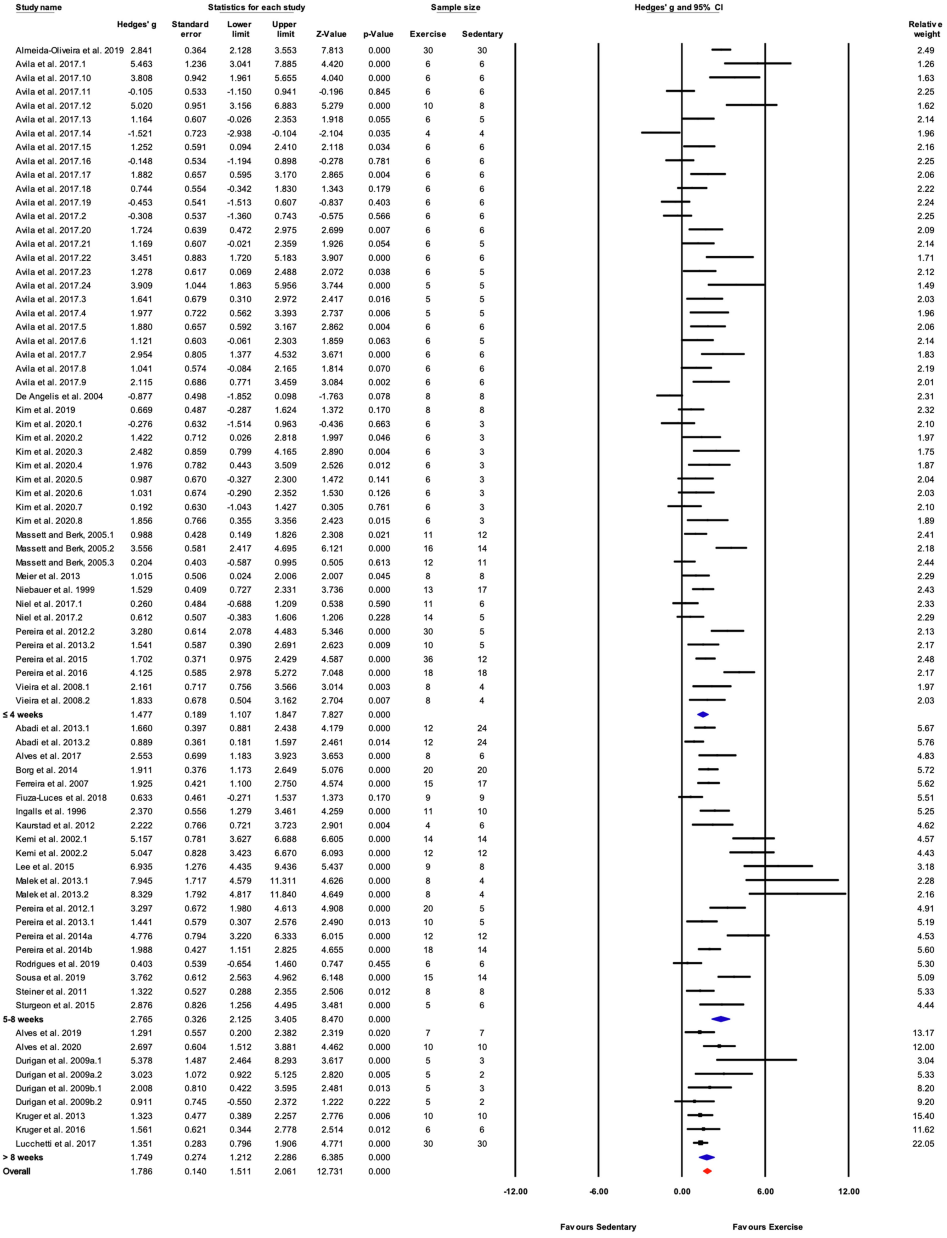
Forest plot of the between-group comparisons of the effect of treadmill incline on performance-based markers of training efficacy. Standardized mean differences were calculated as Hedges’ *g*. Overall analysis was conducted using a random effects model. Values to the left of zero (Favors Sedentary) indicates the sedentary group had a greater response. Values to the right of zero (Favors Exercise) indicates a greater response in the exercise training group. The size of the black squares indicates the weight of the study-specific estimates. Blue diamond indicates pooled estimate of random effects model for each subgroup. Red diamond indicates overall pooled estimate of random effects model.

Studies were divided into three ranges of training duration: “≤4,” “5–8,” and “>8weeks.” Each training duration was associated with a significant increase in performance (Figures 5, 7). Mice training for 5–8weeks had a greater response than those training for a shorter duration (“≤4weeks”), or a longer duration (“>8weeks”; *Q*-between=11.69, df=2, *p*<0.05, *I^2^*=80.3%).

##### Grouped by Exercise Test

**Figure 8:**
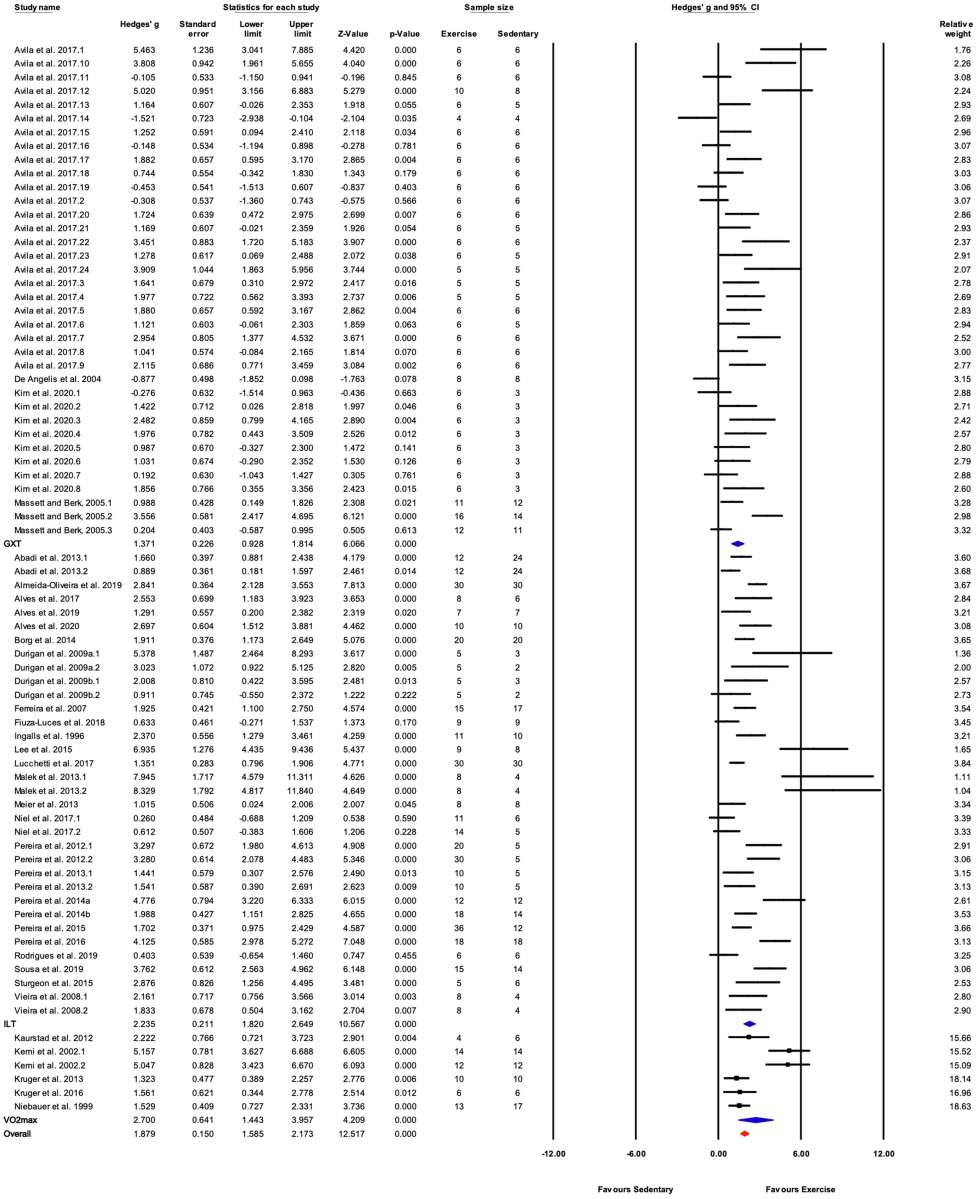
Forest plot of the between-group comparisons of the effect of exercise performance test protocol on performance-based markers of training efficacy. GXT, graded exercise test; ILT, incremental load test; $\dot{V}$O_2max,_ maximal oxygen consumption test. Standardized mean differences were calculated as Hedges’ *g*. FIGURE 8Overall analysis was conducted using a random effects model. Values to the left of zero (Favors Sedentary) indicates the sedentary group had a greater response. Values to the right of zero (Favors Exercise) indicates a greater response in the exercise training group. The size of the black squares indicates the weight of the study-specific estimates. Blue diamond indicates pooled estimate of random effects model for each subgroup. Red diamond indicates overall pooled estimate of random effects model.

There were three subgroups in the covariate exercise test: “GXT,” “ILT,” and “$\dot{V}$O_2max_.” All tests were associated with significant increases in training responses. A significant difference was observed between testing protocols (*Q*-between=9.52, df=2, *p*<0.05, *I^2^*=80.6%; Figures 5, 8). The largest effect of training was observed for studies utilizing the $\dot{V}$O_2max_ test, followed by ILT, and GXT; however, the 95% CI for the $\dot{V}$O_2max_ group included the point estimate of the ILT subgroup. ILT was significantly greater than GXT (Figures 5, 8).

A multivariate meta-regression that included training frequency (day/week), treadmill velocity (m/min) and incline (degrees), time/session (min), and training duration (weeks) was performed to determine the association between exercise training components and performance outcomes. Thirty-five studies were included in the meta-regression. None of the coefficients in a multivariate meta-regression were significant and overall, the model did not explain any of the between-study variance in effect size (*R*^2^=0.0).

##### Results for Biochemical Outcomes

**Figure 9:**
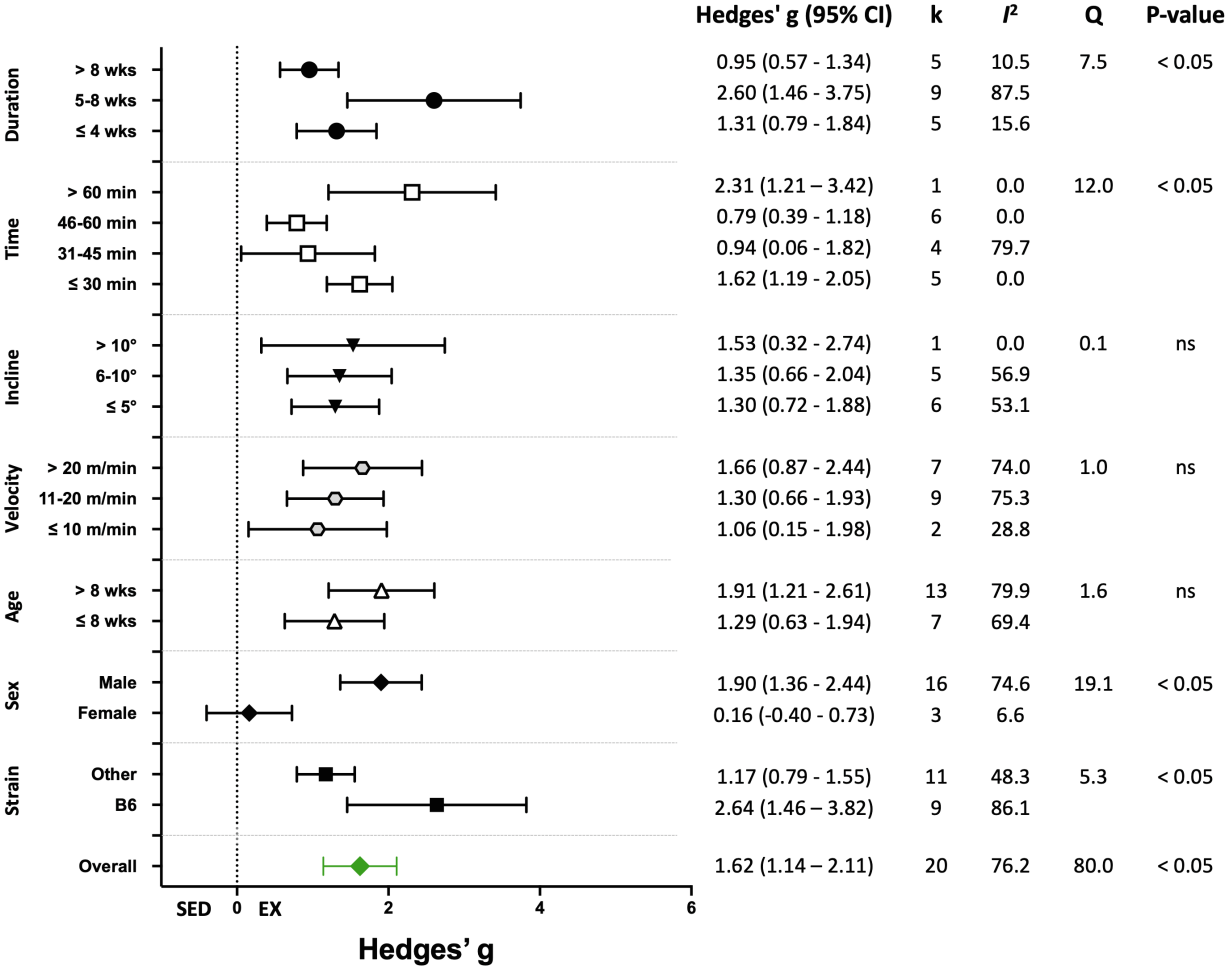
Adjusted effect sizes of the between-group comparisons for the predefined moderators on biochemical exercise training outcomes. GXT, graded exercise test; ILT, incremental load test; $\dot{V}$O_2max_, maximal oxygen consumption test; B6, C57BL/6 mice; k, number of studies in each subgroup; *I*^2^, measure of heterogeneity; *Q*, Cochran’s *Q*; *p*, value of *p* for heterogeneity analysis (overall) or differences between subgroups; ns, non-significant *p*>0.05.

Nineteen percent of the studies (20 of 105) reported biochemical outcomes, including citrate synthase or succinate dehydrogenase activity, or mitochondrial DNA copy number, or lactate levels as the indicators of training efficacy. The overall effect of exercise training on biochemical-based outcome variables was significant, with high heterogeneity (Hedges’ *g*=1.62, 95% CI=1.14–2.11, *p*<0.05, *Q*-value=80.0, df=19, Tau^2^=0.84, *I^2^*=76.2%, prediction interval=−0.37–3.62). A summary of the analyses for the moderator variables analyzed is shown in Figure 9. Significant improvements in biochemical outcomes with exercise training were shown in male mice and in studies with a training duration of 5–8weeks. Significant subgroup differences also were observed for mouse strain and time/session (Figure 9). Results for moderator variables with significant differences between subgroups are described below.

##### Grouped by Mouse Strain

**Figure 10:**
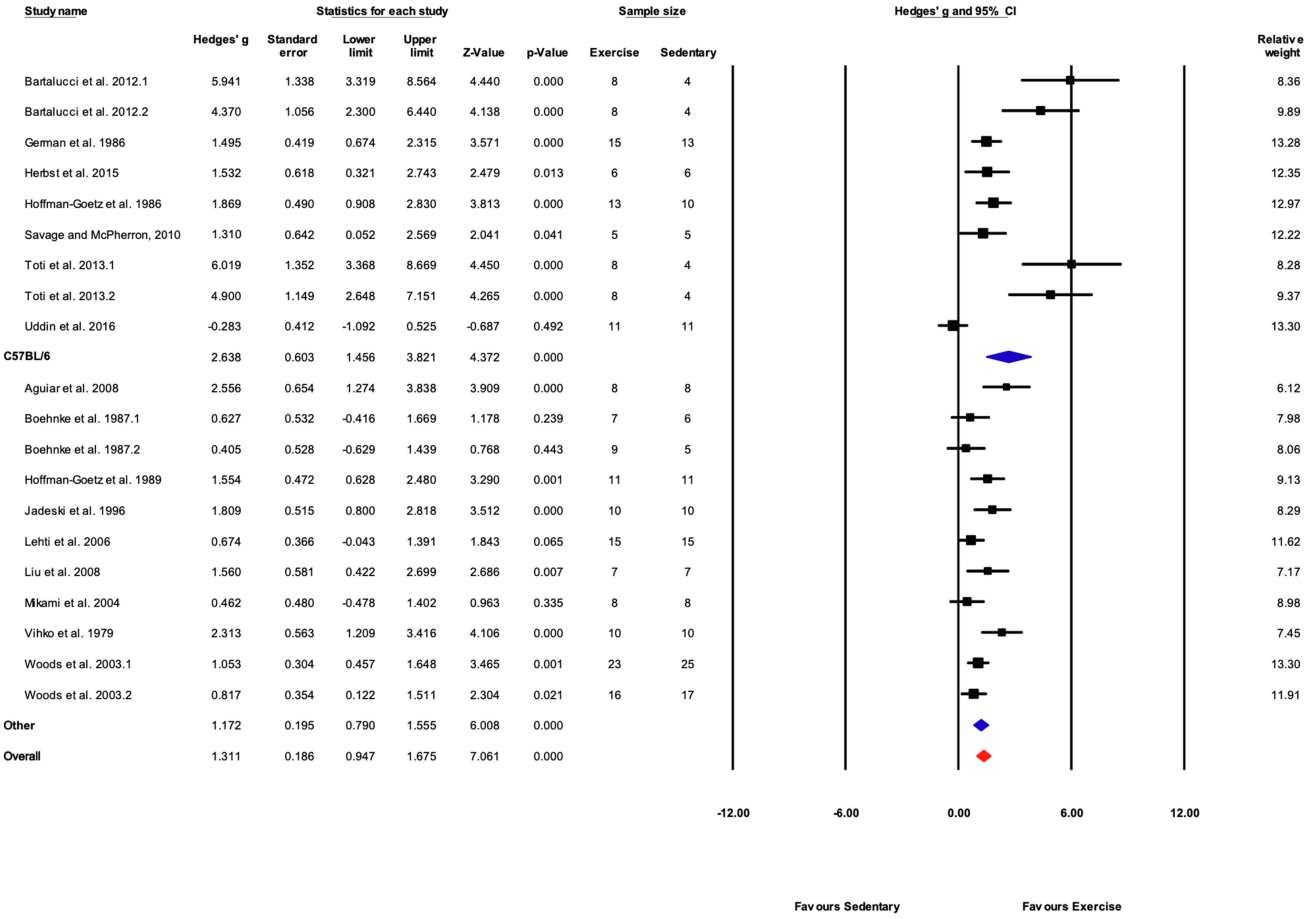
Forest plot of the between-group comparisons of the effect of mouse strain on biochemical trait markers of training efficacy. Standardized mean differences were calculated as Hedges’ *g*. Overall analysis was conducted using a random effects model. Values to the left of zero (Favors Sedentary) indicates the sedentary group had a greater response. Values to the right of zero (Favors Exercise) indicates a greater response in the exercise training group. The size of the black squares indicates the weight of the study-specific estimates. Blue diamond indicates pooled estimate of random effects model for each subgroup. Red diamond indicates overall pooled estimate of random effects model.

Studies were divided into two mouse strain subcategories, C57BL/6 and “Other.” The “Other” category included six mouse strains and accounted for 11 of 20 studies (55%). Both cohorts showed significant responses to training. The response to training was significantly greater in C57BL/6 mice compared with other strains (*Q*-between=5.34, df=1, *p*<0.05, *I^2^*=76.2%; Figures 9, 10).

##### Grouped by Sex

**Figure 11:**
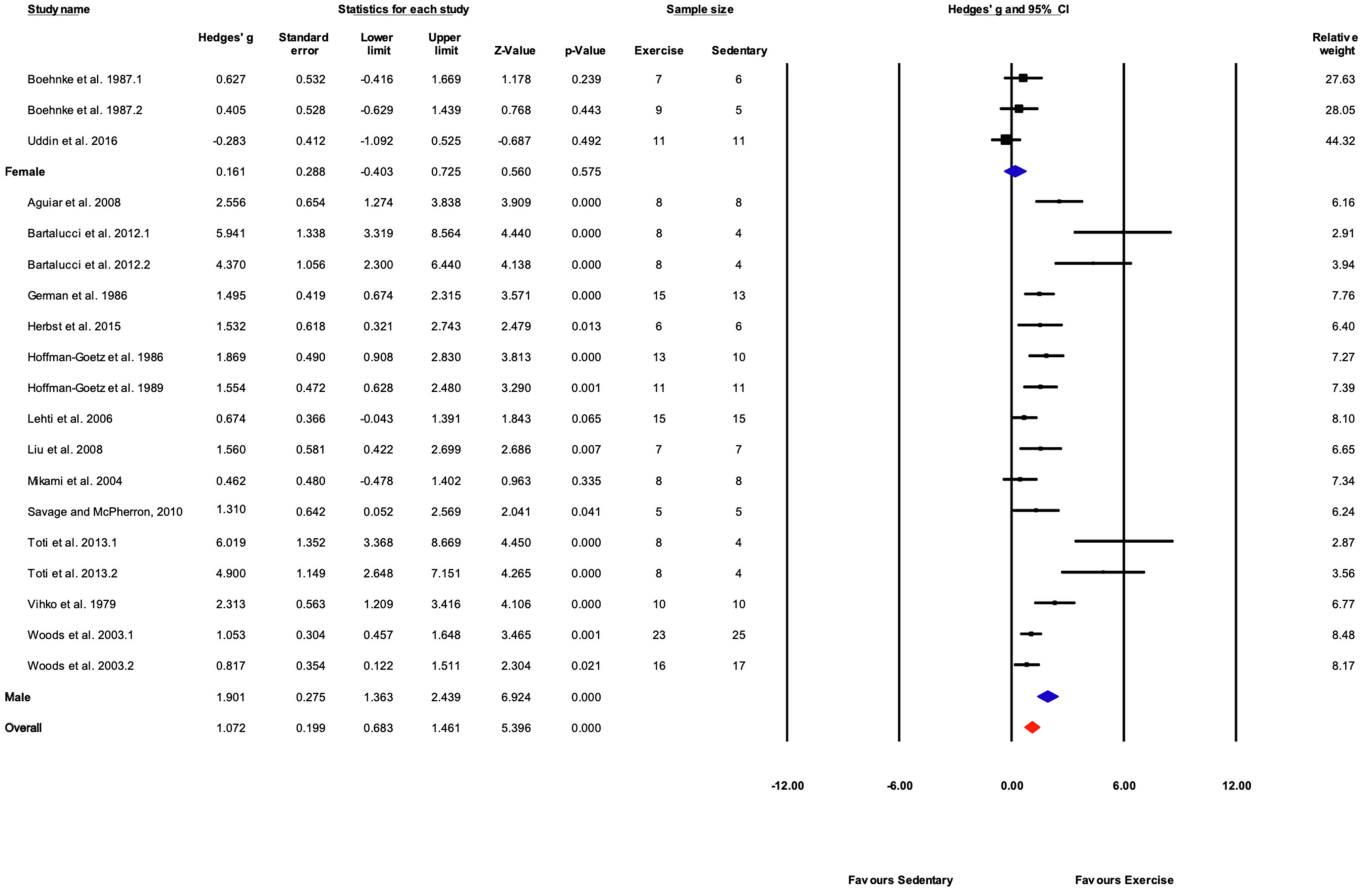
Forest plot of the between-group comparisons of the effect of sex on biochemical trait markers of training efficacy. Standardized mean differences were calculated as Hedges’ *g*. Overall analysis was conducted using a random effects model. Values to the left of zero (Favors Sedentary) indicates the sedentary group had a greater response. Values to the right of zero (Favors Exercise) indicates a greater response in the exercise training group. The size of the black squares indicates the weight of the study-specific estimates. Blue diamond indicates pooled estimate of random effects model for each subgroup. Red diamond indicates overall pooled estimate of random effects model.

Subgroup analysis shows a significant difference between male and female mice (*Q*-between=19.1, df=1, *p*<0.05, *I^2^*=77.1%). Only three studies included female mice compared with 16 using male mice. Female mice showed a non-significant response to training (*p*>0.05) compared with sedentary controls (Figures 9, 11).

##### Grouped by Time

**Figure 12:**
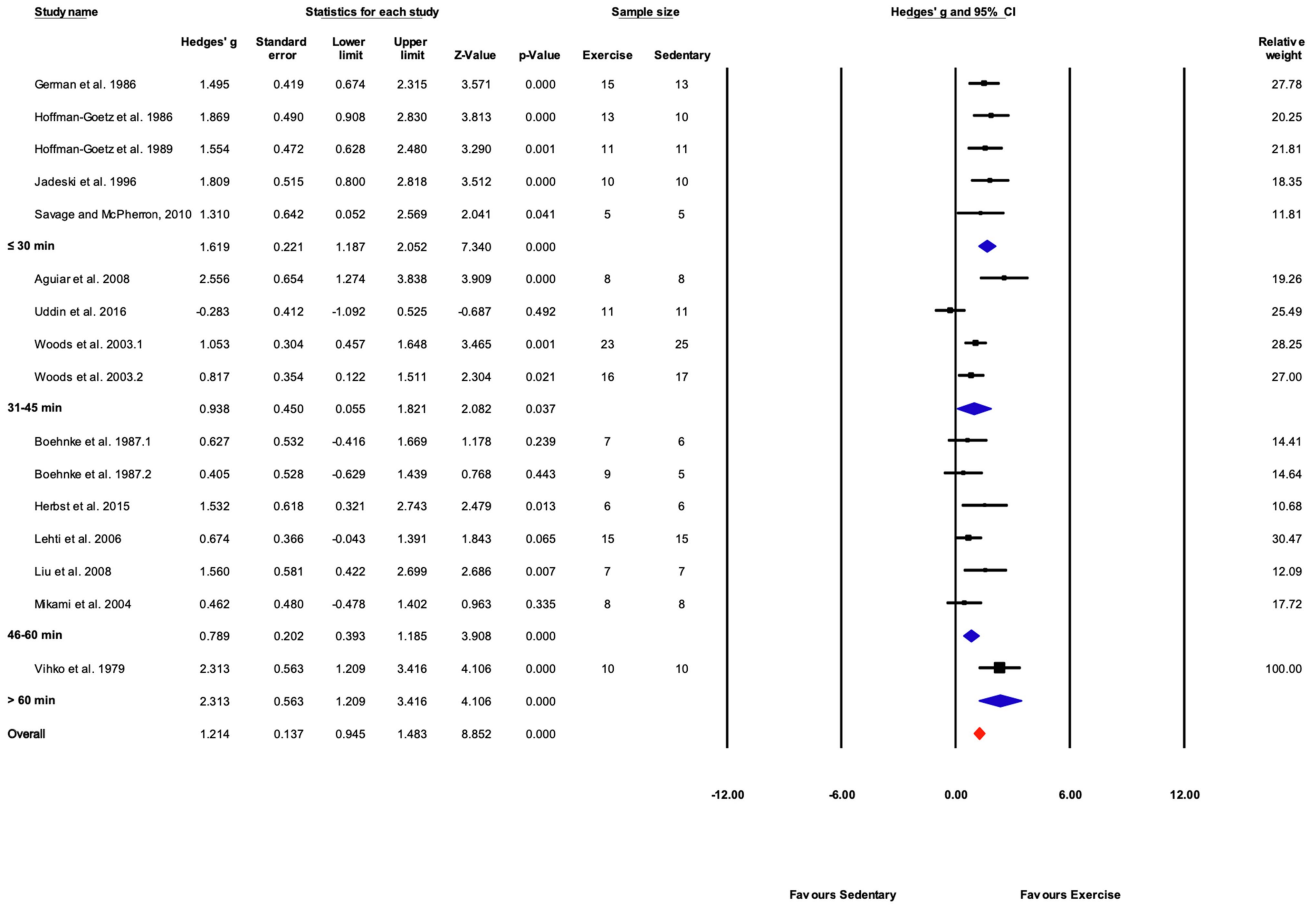
Forest plot of the between-group comparisons of the effect of exercise training session time on biochemical trait markers of training efficacy. Standardized mean differences were calculated as Hedges’ *g*. Overall analysis was conducted using a random effects model. Values to the left of zero (Favors Sedentary) indicates the sedentary group had a greater response. Values to the right of zero (Favors Exercise) indicates a greater response in the exercise training group. The size of the black squares indicates the weight of the study-specific estimates. Blue diamond indicates pooled estimate of random effects model for each subgroup. Red diamond indicates overall pooled estimate of random effects model.

Exercise time per session was divided into four subgroups: “≤30,” “31–45,” “46–60,” and “>60min” consisting of 5, 4, 6, and 1 studies, respectively. Exercise training elicited a significant effect in each exercise time subgroup. Significant differences between subgroups were present (*Q*-between=11.99, df=3, *p*<0.05, *I^2^*=56.6%). The largest effect was in the one study in the “>60min” subcategory, followed by the “≤30min” subcategory (Figures 9, 12).

##### Grouped by Training Duration

**Figure 13:**
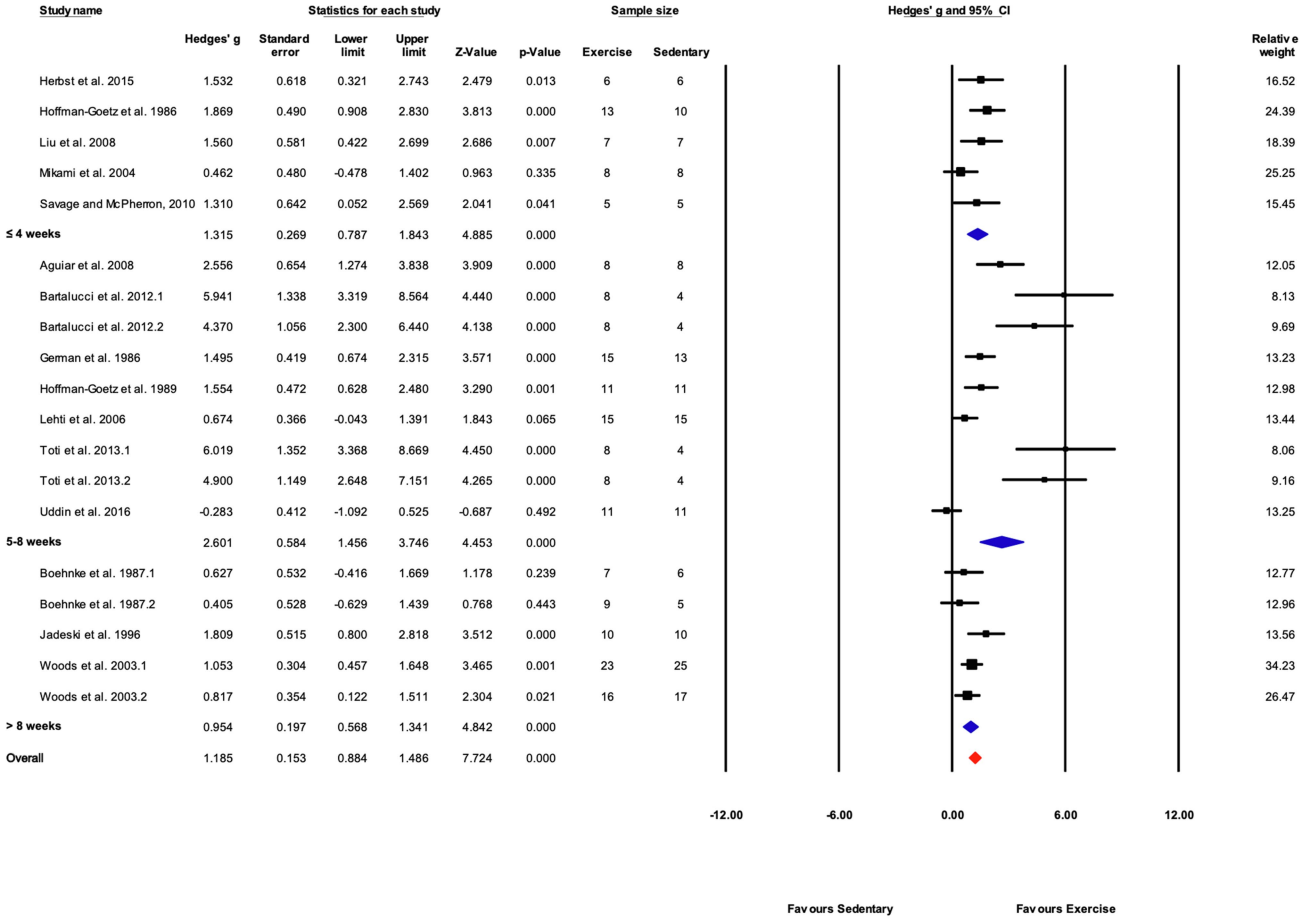
Forest plot of the between-group comparisons of the effect of training protocol duration on biochemical trait markers of training efficacy. Standardized mean differences were calculated as Hedges’ *g*. Overall analysis was conducted using a random effects model. Values to the left of zero (Favors Sedentary) indicates the sedentary group had a greater response. Values to the right of zero (Favors Exercise) indicates a greater response in the exercise training group. The size of the black squares indicates the weight of the study-specific estimates. Blue diamond indicates pooled estimate of random effects model for each subgroup. Red diamond indicates overall pooled estimate of random effects model.

As in the overall and performance-based outcome analyses, the effect of exercise training was significant for all training durations (Figures 9, 13). The response to training was significantly greater in the “5–8weeks” group compared with “≤4” and “>8weeks” (*Q*-between=7.48, df=2, *p*<0.05, *I^2^*=76.4%).

A multivariate meta-regression that included training frequency (day/week), treadmill velocity (m/min) and incline (degrees), time/session (min), and training duration (weeks) was performed to determine the association between exercise training components and biochemical outcomes. Eleven studies had complete data for each variable and were included in the meta-regression analysis. Although none of the coefficients in the model were significant, 100% of the between-study variance was explained by the model (*R*^2^=1.00).

## Discussion

The main findings of this systematic review and meta-analysis of mouse exercise training studies are: (1) a relatively small number of studies incorporating exercise training report a “classical” measure of training efficacy; (2) many studies do not report complete information regarding the exercise training protocol; (3) the majority of exercise training studies utilize male mice only; (4) exercise training significantly increases measures of training efficacy; and (5) exercise prescription parameters do not explain a significant amount of variation between studies when changes in exercise performance are used as a marker for training efficacy.

Our systematic review identified 164 full-text articles that included a treadmill training protocol with untreated mice assigned to either a sedentary control group or exercise training group. Of these, approximately 35% included a “classical” marker of training efficacy. Increases in skeletal muscle enzyme activity, mitochondrial DNA, and/or changes in skeletal muscle fiber types are possible markers for adaptations to endurance exercise training (Booth et al., 2010). An increase in peak or maximal oxygen consumption is often considered the gold standard in human-based endurance exercise training studies. In animal studies, changes in exercise performance are typically used as a surrogate for maximal oxygen consumption (Fuller and Thyfault, 2021). Therefore, only studies including these or other well-known markers for exercise training adaptations were included (Holloszy and Coyle, 1984; Hellsten and Nyberg, 2015). The majority of studies that were excluded for lack of such a marker utilized body weight differences between sedentary and exercise-trained groups as a general marker for exercise training. Although lower body weights in the exercise training group might be related to increased physical activity, body weight differences alone do not necessarily indicate that the exercise training elicited beneficial biochemical and/or cardiorespiratory fitness adaptations. For purposes of replication and thorough analysis of the responses to exercise, exercise training studies should include all relevant information regarding the training protocol such as frequency, intensity, and duration (Booth et al., 2010). All protocol information was included in 48% of the studies. Treadmill velocity (38%) and incline (39%) were the most frequently omitted variables. Most reported frequency, session time and duration. Exercise intensity was reported in 68% of studies, but the basis for qualifiers low, moderate, and high were unclear. Treadmill velocity and incline were frequently omitted when exercise intensity as a percentage of maximum was reported. Collectively, these results indicate that treadmill-based exercise training studies in mice frequently do not report all the components of the exercise training program or well-accepted adaptations to exercise training as indicators of training efficacy.

Mouse strain, sex, and age have been reported to influence exercise training responses. Overall, these moderators had limited effects on exercise training responses. When outcome variables were divided into performance-based and biochemical outcomes, sex and mouse strain significantly influenced biochemical responses to training (Figure 9). Male mice had significantly greater biochemical adaptations to exercise training than female mice. In contrast, performance-based outcomes were somewhat greater in females than males, but not significantly so (Figure 5). In a direct comparison, Kemi et al. (2002) reported that $\dot{V}$O_2max_ was significantly greater in trained female mice than in similarly trained male mice. Similarly, exercise training-induced cardiac hypertrophy was greater in female mice compared with males (Foryst-Ludwig et al., 2011). However, less than 10% of the included studies utilized female mice and only three full-text articles included both male and female mice (Kemi et al., 2002; Foryst-Ludwig et al., 2011; Abadi et al., 2013). Therefore, additional studies are needed investigating the responses to endurance exercise training in female mice as well as studies directly comparing responses in mice of both sexes.

The influence of mouse strain was not significant overall (Table 2), but was significant in studies measuring biochemical markers of exercise training. For subgroup analyses, strains were coded as C57BL/6 or “Other.” The “Other” group included data from 27 strains. As with sex comparisons, only three articles included data from multiple mouse strains (Massett and Berk, 2005; Avila et al., 2017; Kim et al., 2020). Each of those publications reported significant strain-dependent changes in exercise capacity in response to exercise training. However, those findings were not supported by the results of the current study for performance-based outcomes. One possible explanation for this disparity is the “Other” strain category is composed of too many individual strains, leading to a high level of variation across subject populations and training protocols. However, the precision and dispersion of the effect estimates are similar for both C57BL/6 and “Other” subgroups suggesting that the variability in response to training is comparable. Thus, the strain-dependent differences in changes in exercise capacity with exercise training reported by Massett and colleagues (Massett and Berk, 2005; Avila et al., 2017; Kim et al., 2020) might be specific to the exercise training and testing paradigm used in those studies. Each of those studies utilized similar exercise training parameters with some strain-specific adjustments which facilitated direct comparisons with minimal variation between training protocols. Conversely, responses in C57BL/6 mice were significantly greater than other strains for biochemical markers of exercise training. This result implies that C57BL/6 mice show greater biochemical adaptations to exercise training than mice from other strains. This contrasts with performance-based outcomes where C57BL/6 mice have low to moderate responses to training compared with other strains (Massett and Berk, 2005; Avila et al., 2017; Kim et al., 2020). Future research investigating the effect of mouse strain on exercise training responses should consider including multiple strains within the same study design and measuring both performance-based and biochemical markers of training efficacy. Collectively, the findings regarding the contribution of sex and mouse strain on responses to exercise training suggest that direct comparisons within a given experimental design might yield results different from those obtained in a pooled analysis of the published studies utilizing individual mouse strains.

Overall, exercise training elicited significant increases in exercise training-associated outcomes. Heterogeneity was high for the combined analysis as well as for outcome-specific analyses. Therefore, subgroup analyses were performed for the combined data and for performance and biochemical outcomes separately. In the combined analysis, the greatest percentage of variation in the effect size was explained by exercise test protocol subgroups (Table 2). Exercise training parameters of frequency, treadmill velocity and incline, exercise session time, and training duration also were investigated to determine their contribution to the heterogeneity between studies/as potential moderator variables. In the overall analysis of 105 studies and in the separate analyses based on training outcome, subgroup analysis was significant for training duration (Table 2; Figures 5, 9). Studies utilizing a training duration of 5–8weeks had significantly greater outcomes than those incorporating longer or shorter periods. Typically, exercise training protocols include one or more weeks during which training time/intensity is progressively increased until the target parameters are reached. For studies ≤4weeks, the target workload might be sustained for too short a period (e.g., 2weeks) to elicit maximal training responses. Protocols longer than 8weeks showed effects comparable to those ≤4weeks, implying that longer duration training protocols might hinder adaptations to training. The mechanism for this is unclear, but declines in performance with prolonged training, especially at higher intensities, can be associated with overtraining syndrome (Pereira et al., 2012; Meeusen et al., 2013). These results suggest that regardless of training outcome a training duration of 5–8weeks appears optimal for adaptations to treadmill training in mice.

Significant subgroup differences for treadmill incline were observed in the combined analysis and for performance-based outcomes (Table 2; Figures 5, 6). Exercise training protocols utilizing an incline above 10° had the largest effect on training outcomes in the combined and performance-based analyses (Table 2; Figures 5, 6). The higher incline should require more work/greater effort and therefore, might elicit greater adaptations to training (Hoydal et al., 2007; Poole et al., 2020). Kemi and colleagues (Kemi et al., 2002; Hoydal et al., 2007) reported that the best estimates of mouse $\dot{V}$O_2max_ are obtained during treadmill exercise at inclines between 15° and 35°. They demonstrated significant improvements in maximal oxygen consumption after 8weeks of training at 25° in male and female C57BL/6J mice (Kemi et al., 2002). Therefore, they recommended an inclination of 25° as optimal for exercise training. However, Petrosino et al. (2016) limited the treadmill incline to 15° in the development of their exercise testing protocol because they observed that mice had difficulty maintaining gait at inclinations above 15°. Although gait changes during treadmill running in rodents, including raising of the snout and lowering the hindquarters, can occur prior to exhaustion (Copp et al., 2009), it is unclear if similar gait changes occur at higher treadmill inclinations in the absence of fatigue. Only five studies utilized an incline >10° (Kemi et al., 2002; Kaurstad et al., 2012; Herbst et al., 2015; Alves et al., 2020) and no direct comparisons of treadmill incline on training responses were included in those articles. Therefore, additional research is required to confirm that treadmill inclination above 10° should be utilized for exercise training programs.

The other exercise training parameter showing subgroup differences was exercise time/session for biochemical-based outcomes. Exercise time per session varied from 30 to 90min in the biochemical outcome group. One study with an exercise time >60 and a biochemical outcome showed a very large effect of exercise training (Vihko et al., 1979), but a wide 95% CI. Exercise times ≤30min had a greater effect on training responses than those with training session times between 31 and 60min. The effect size for the ≤30min subgroup also was the largest in the combined group analysis, however, there were no significant subgroup differences between exercise times in the overall analyses. Given the relatively small number of studies per subgroup, additional research is required to confirm that shorter exercise training sessions might elicit greater biochemical training adaptations than longer individual exercise sessions.

Significant subgroup differences were observed for the exercise test used to assess changes in performance. The three most common testing protocols were increasing treadmill velocity at a fixed incline (ILT), increasing both treadmill velocity and incline at fixed intervals (GXT), and tests measuring maximal oxygen consumption ($\dot{V}$O_2max_). Time or distance were typically used to assess performance in the ILT and GXT tests. The largest effect of exercise training was observed for $\dot{V}$O_2max_ tests (Figures 5, 8). This subgroup included six studies and had a relatively wide 95% CI (1.44–3.96). The testing protocols used to measure mouse $\dot{V}$O_2max_ are like the protocols for ILT, increasing speed at a constant incline. But the criteria for reaching $\dot{V}$O_2max_, e.g., a plateau in $\dot{V}$O_2_ with increasing workload and respiratory exchange ratio above 1.0, is more clearly defined than those for ILT and GXT tests (e.g., time touching the shock grid or number of shocks; Poole et al., 2020). Therefore, $\dot{V}$O_2max_ tests were placed in a separate category from ILT. The effect size for ILT was greater than that for GXT. The GXTs were primarily used by one group (Massett and Berk, 2005; Avila et al., 2017; Kim et al., 2020) and all the study protocols were 4weeks in length. In contrast, average training duration in studies utilizing ILT protocols was 7.4±2.8weeks. Shorter duration exercise training was associated with smaller responses to exercise training (Figures 5, 9) and thus, might explain some of the differences between studies utilizing ILT vs. GXT protocols. It is unclear whether differences between GXT and ILT test protocols would be observed if training programs were matched for duration. Furthermore, the combination of increasing treadmill incline and speed throughout the GXT test results in larger increases in exercise intensity at specific stages. In humans, GXT-type tests result in less uniform increases in physiological responses and more variable estimates of exercise capacity and/or oxygen consumption (Myers et al., 1991; Pescatello et al., 2014). Similar results in mice might lead to inaccurate exercise prescription and subsequently less than optimal responses to exercise training (Hoydal et al., 2007).

To further explore the contribution of moderator variables on exercise training effects, meta-regression was used to determine the role of individual variables as well as multiple variables on variation across studies. The models tested included frequency of training, treadmill velocity and incline, time per session, and training study duration to identify the exercise prescription variables most related to exercise training outcomes. The model including all these variables accounted for 0% of between-study variance when all studies were considered. When studies were divided by outcome variables, this same model did not explain any of the between-study variance for performance-based outcomes (*R*^2^=0.0). In contrast, this same model explained 100% of the variance for studies reporting a biochemical outcome for exercise training despite no individual variable having a *p*<0.05. The general recommendation for meta-regression is that 10 studies should be included for each moderator variable (Baker et al., 2009). The number of studies included in the meta-regression analysis for all studies met this recommendation. However, the number of studies included with biochemical outcomes was small and therefore, the strength of the association should be interpreted with caution. Nevertheless, these results suggest that biochemical outcome variables are more strongly related to exercise training program components than are performance-based outcomes. This association implies that biochemical measurements should be incorporated into exercise training studies to provide evidence of training efficacy. This recommendation was proposed previously (Booth et al., 2010) but comes with the caveat that many of these measurements are invasive and require terminal procedures (Handschin et al., 2010).

The measurement of exercise performance in mice, including $\dot{V}$O_2max_, is somewhat controversial. Versions of different protocols for measuring $\dot{V}$O_2max_ in mice have been proposed in the literature, each with varying levels of evidence to support the protocol (Kemi et al., 2002; Marcaletti et al., 2011; Ayachi et al., 2016; Petrosino et al., 2016; Lemaire et al., 2017). In addition, the validity of surrogates for $\dot{V}$O_2max_ (e.g., time to exhaustion) as estimates of exercise capacity have been questioned because of the subjective nature of the definition of volitional fatigue and/or exhaustion (Booth et al., 2010; Fuller and Thyfault, 2021) and issues with repeatability (Knab et al., 2009). Knab et al. (2009) speculated that repeatability of exercise performance measures during a maximal exercise test in mice might be related to the outcome variable and the investigator’s definition of maximum. In contrast, biochemical outcomes are laboratory-based measurements with quantitative outcomes which might lead to less subjective interpretation of the outcome variable. Although some variation is likely associated with biochemical markers (Lonbro et al., 2019), standardized measurement procedures could reduce intra- and inter-investigator variation. Therefore, changes in these variables might demonstrate more consistent responses to a specific exercise intervention.

### Limitations

Although data from 10 moderator variables were extracted and analyzed to explain heterogeneity between studies, there are several other factors that might influence exercise training responses. Housing temperature and time of day have been shown to influence responses to exercise and adaptations to training (Wolff and Esser, 2012; McKie et al., 2019; Sato et al., 2019). Information regarding these variables were not included as part of the data extraction process. Interest in the effect of these environmental variables on responses to exercise training is growing and subsequent analyses should consider these moderator variables. In addition, many training studies include one or more weeks of progressive increases in training load to attain a final target workload. In the current study, only the final target workload was considered for analyses. Although this early phase of the training program might influence the overall outcome, this phase was generally not well described and difficult to quantify for analytical purposes and was therefore not analyzed as part of the training program. Finally, a few studies reported subject characteristics or training paradigms as ranges. In these cases, the median value was used for any moderator variables reported as ranges to minimize missing data for any given study.

In conclusion, the results of this systematic review and meta-analysis demonstrate there is a high degree of heterogeneity across endurance exercise training studies in mice. Training duration had a significant effect of training outcome, whether the outcome was performance-based or related to biochemical traits. Parameters for exercise training prescription explained a small percentage of the variation in outcomes for performance-based traits. Therefore, investigators should consider measuring both performance and biochemical outcomes to confirm training efficacy. In addition, the lack of data on training adaptations in female mice suggests that future studies should include both male and female mice or focus solely on responses in female mice to better understand the effect of sex on exercise training responses.

## Data Availability Statement

The raw data supporting the conclusions of this article will be made available by the authors, without undue reservation.

## Author Contributions

HK, CM, and MM reviewed the abstracts, titles, and full text, extracted and reviewed the data, and drafted, edited, and revised the manuscript. MM analyzed the data. All authors contributed to the article and approved the submitted version.

## Funding

This work was supported by funds from the Texas Tech University Office of Vice President for Research.

## Conflict of Interest

The authors declare that the research was conducted in the absence of any commercial or financial relationships that could be construed as a potential conflict of interest.

## Publisher’s Note

All claims expressed in this article are solely those of the authors and do not necessarily represent those of their affiliated organizations, or those of the publisher, the editors and the reviewers. Any product that may be evaluated in this article, or claim that may be made by its manufacturer, is not guaranteed or endorsed by the publisher.

## Footnotes

^1^ https://automeris.io/WebPlotDigitizer/

^2^ www.Meta-Analysis.com/Prediction
